# Early and Late Processes Driving NET Formation, and the Autocrine/Paracrine Role of Endogenous RAGE Ligands

**DOI:** 10.3389/fimmu.2021.675315

**Published:** 2021-09-20

**Authors:** Olga Tatsiy, Vanessa de Carvalho Oliveira, Hugo Tshivuadi Mosha, Patrick P. McDonald

**Affiliations:** ^1^Pulmonary Division, Faculty of Medicine, Université de Sherbrooke, Sherbrooke, QC, Canada; ^2^Centre de recherche du CHUS (CRCHUS), Sherbrooke, QC, Canada; ^3^Department of Immunology and Cell Biology, Faculty of Medicine, Université de Sherbrooke, Sherbrooke, QC, Canada

**Keywords:** neutrophils, extracellular traps, signaling, protein arginine deiminase, chromatin decondensation, autocrine, RAGE (receptor for advanced glycation end products)

## Abstract

Neutrophil extracellular trap (NET) formation has emerged as an important response against various pathogens; it also plays a role in chronic inflammation, autoimmunity, and cancer. Despite a growing understanding of the mechanisms underlying NET formation, much remains to be elucidated. We previously showed that in human neutrophils activated with different classes of physiological stimuli, NET formation features both early and late events that are controlled by discrete signaling pathways. However, the nature of these events has remained elusive. We now report that PAD4 inhibition only affects the early phase of NET generation, as do distinct signaling intermediates (TAK1, MEK, p38 MAPK). Accordingly, the inducible citrullination of residue R2 on histone H3 is an early neutrophil response that is regulated by these kinases; other arginine residues on histones H3 and H4 do not seem to be citrullinated. Conversely, elastase blockade did not affect NET formation by several physiological stimuli, though it did so in PMA-activated cells. Among belated events in NET formation, we found that chromatin decondensation is impaired by the inhibition of signaling pathways controlling both early and late stages of the phenomenon. In addition to chromatin decondensation, other late processes were uncovered. For instance, unstimulated neutrophils can condition themselves to be poised for rapid NET induction. Similarly, activated neutrophils release endogenous proteic factors that promote and largely mediate NET generation. Several such factors are known RAGE ligands and accordingly, RAGE inbibition largely prevents both NET formation and the conditioning of neutrophils to rapidly generate NETs upon stimulation. Our data shed new light on the cellular processes underlying NET formation, and unveil unsuspected facets of the phenomenon that could serve as therapeutic targets. In view of the involvement of NETs in both homeostasis and several pathologies, our findings are of broad relevance.

## Introduction

Neutrophil extracellular traps (NETs) consist of extruded chromatin adorned with histones, proteases, and several other components, which immobilize pathogens and participate in their killing. In addition to representing an important antimicrobial response, NETs also influence disease progression in chronic inflammation, autoimmunity, and cancer (1). NETs have additionally been shown to promote inflammation resolution through the proteolysis of cytokines and chemokines (2). NET formation is understood to involve several steps, at least in cells stimulated with PMA or monosodium urate crystals (3, 4). Under these conditions, elastase was shown to translocate from azurophil granules to the nucleus, where it is thought to partially cleave histones, aiding in chromatin decondensation. Myeloperoxidase similarly escapes cytoplasmic granules, enters the nucleus, and binds to chromatin in the late stages of the process to promote further decondensation (3). This eventually leads to nuclear swelling, and there is evidence for a role of LL-37 (a specific granule component) in causing nuclear membrane rupture (5). The entire process culminates with chromatin extrusion into the extracellular space. Thus, a general picture of the events underlying NET formation has started to emerge.

Up until recently, our understanding of the signaling pathways controlling NET formation was fragmented and incomplete. While Syk and PI3K seemed to stand out as crucial intermediates for NET generation in response to several neutrophil stimuli (6–10), a role for other signaling pathways (e.g. p38 MAPK, MEK, JNK, PKC) had also been described, albeit with the caveat that the latter studies usually consisted of isolated observations for a given pathway, using different stimuli and often different methods (6, 8, 11, 12). In two recent articles, we revisited the issue of signaling components affecting NET generation using an assay that is specific for extruded chromatin and standardized for cell number, and by systematically comparing several classes of physiological neutrophil stimuli (inflammatory cytokines, chemoattractants, growth factors, and inflammatory microcrystals) (13, 14). For all stimuli investigated, we confirmed the paramount importance of the Syk and PI3K pathways for NET formation, and further established that they affect late stages of the phenomenon (i.e. 90 min or more post-stimulation) (13, 14). Using the same integrated approach, we showed that TAK1, p38 MAPK, and MEK profoundly affect NET generation, but by acting on immediate/early events, i.e. within the first 30 min of stimulation (13, 14). By contrast, we found no involvement of the Src, PKC, or JNK pathways (13, 14). Thus, there appear to be common signaling components controlling NET formation that are shared across several classes of physiological NET inducers, and these pathways affect either early or late events.

The nature of these early and late events has yet to be determined. Because hours are required for chromatin extrusion (between 2 and 4 h, depending on the experimental conditions), and because several neutrophil products that act as NET inducers can be secreted during this time frame in stimulated cells (e.g. inflammatory cytokines and chemokines), we and others investigated whether the inhibition of gene transcription or protein synthesis might interfere with NET formation. However, neither process was found to be involved (13, 15, 16). One group obtained divergent results by reporting that transcription contributes to NET production, but they nonetheless found that protein synthesis did not (17). Thus, while a contribution of *de novo*-synthesized proteins to NET formation can be ruled out, the phenomenon could still involve pre-stored products, as reported for intracellular elastase and LL-37 (3, 5). In this study, we report that unstimulated neutrophils can condition themselves to be poised for rapid NET production, and that activated neutrophils release endogenous factors that promote this response. Both processes represent late events in NET formation. Another late event is chromatin decondensation, which is controlled by most signaling pathways known to affect NET formation. Conversely, PAD4 inhibition only affects the early phase of NET generation, and accordingly, the citrullination of histone H3 on its R2 residue is an early neutrophil response that is regulated by TAK1, MEK, and p38 MAPK.

## Materials and Methods

### Antibodies and Reagents

Antibodies against citrullinated histone H3 and H4 were from Abcam (ab176843, ab219406, ab219407, ab81797); phospho antibodies were all from Cell Signaling (Beverly, MA, USA). Ficoll-Paque Plus was from GE Biosciences (Baie d’Urfé, Qc, Canada); endotoxin-free (< 2 pg/ml) RPMI 1640 was from Wisent (St-Bruno, Qc, Canada). Recombinant human cytokines were from R&D Systems (Minneapolis, MN, USA). Monosodium urate crystals (MSU) were from Cayman Chemical (Ann Arbour, MI, USA). N-formyl-methionyl-phenylalanine (fMLP) and phenylmethanesulphonyl fluoride (PMSF) were from Sigma (St. Louis, MO, USA). All inhibitors, antagonists, and fluorescent probes were purchased through Cedarlane Labs (Missisauga, Canada). PlaNET reagents (fluorescent chromatin-binding polymers) were from Immune Biosolutions (https://immunebiosolutions.com/en/pipeline/planet-reagents/).

### Cell Isolation and Culture

Neutrophils were isolated from the peripheral blood of healthy donors, under a protocol approved by an institutional ethics committee (Comité d’éthique de la recherche du CIUSS de l’Estrie-CHUS). All subjects gave written informed consent in accordance with the Declaration of Helsinki. Briefly, whole blood was collected using an anticoagulant (sodium citrate), and successively submitted to dextran sedimentation, Ficoll separation, and water lysis – as previously described (18). The entire procedure was carried out at room temperature under endotoxin-free conditions. As determined by Wright staining and FACS analysis, final neutrophil suspensions contained fewer than 0.1% monocytes or lymphocytes; neutrophil viability exceeded 98% after 4 h in culture, as determined by trypan blue exclusion and by Annexin V/propidium iodide FACS analysis.

### NET Assays

For each condition, 500 µl of a neutrophil suspension (2x10^6^/ml in RPMI 1640/2% autologous serum) was deposited onto coverslips that had been freshly coated with poly-L-lysine and placed inside the wells of a 24-well plate; the cells were then left to adhere for 60 min in a cell culture incubator. Cells were gently washed with pre-warmed culture medium and covered with 500 µl of fresh, pre-warmed medium. Inhibitors and/or stimuli were then added, and the final volume brought to 550 µl, prior to a 4-h incubation (37°C, 5% CO2). Reactions were stopped by adding 500 µl ice-cold PBS containing 1 mM PMSF, and the coverslips were placed on ice for 10 min. The liquid on the coverslips was discarded and cells were incubated (90 min on ice, with gentle shaking) in 1 ml of PBS containing 1 mM PMSF and diluted PlaNET reagent (as recommended by the manufacturer). Cells were finally fixed (15 min, room temperature) in PBS containing 2% parafornaldehyde, as well as a nuclear stain. The fixed cells were then washed with PBS, and the coverslips mounted onto glass slides using a drop of mounting medium (ProLong Gold, Life Technologies), prior to epifluorescence microscopy analysis. For quantitation, 3 fields at 10x magnification were typically counted, that never included the coverslip edges: this amounts to about 1,000 neutrophils per experimental condition in each experiment.

In some experiments, neutrophil supernatants were collected to assess their ability to induce NET formation. In the case of unstimulated or MSU-activated cells, the supernatants were spun (18,000 g, 10 min, 4°C) to pellet the MSU crystals and any cells that might have detached during collection. In the case of TNF-activated neutrophils, supernatants were incubated (2 h, room temperature, on a rotator wheel) with a neutralizing Ab (Peprotech #500-M26, 0.5 µg/ml final concentration) and further incubated (2 h, room temperature, on a rotator wheel) with protein G-sepharose beads, prior to centrifugation (18,000 g, 10 min, 4°C). In some other experiments, stimulus-depleted supernatants (or supernatants from unstimulated cells) were digested with 30 U/ml proteinase K for 3h at 37°C. The enzyme was then inactivated by adding PMSF (to 0.1 mM, final concentration).

### Nuclear Decondensation Analyses

Neutrophils cultured on poly-L-lysine-coated coverslips were placed at 37°C under a humidified 5% CO2 atmosphere in the presence or absence of inhibitors or stimuli, as described. After 3 h, Hoechst 33342 and propidium iodide (2 µM and 5 µM final concentrations, respectively) were added to the culture medium and the cells were placed in the temperature-controlled chamber of a confocal microscope. The cells were further incubated for 30 min. Cells with large, rounded nuclei (as opposed to polylobed nuclei) were counted as those which underwent chromatin decondensation. For this purpose, 3 fields at 40x magnification were typically counted: this amounts to about 400 neutrophils per experimental condition in each experiment.

### Immunoblots

Neutrophils were made to adhere to coverslips coated with poly-L-lysine and cultured as described above in 6-well plates; reactions were stopped by removing the culture medium, placing the culture plates on an ice bed, and adding ice-cold PBS containing protease inhibitors, as previously described (19, 20). Cells were gently scraped, collected, and centrifuged (2000 g, 5 min, 4°C); the resulting pellets were resuspended in boiling sample buffer and then incubated 5 min at 95°C. Samples were electrophoresed, transferred onto nitrocellulose, and processed for immunoblot analysis as previously described (19, 20).

### Mass Spectrometry Proteomics Analyses

Neutrophils were incubated as described above for NET assays but in the absence of serum to avoid an overabundance of seric proteins in the subsequent MS analyses. Culture supernatants were collected in low adsorption eppendorf tubes, incubated with TNF neutralizing antibodies (2 h, 4°C, on a rotator), further incubated with protein G-Sepharose 4FF beads (30 min, 4°C, on a rotator), and spun (18,000 g, 15 min, 4°C) to immunodeplete the stimulus and pellet any cells that might have detached during culture. The resulting supernatants were processed for peptide preparation and purification as described before (21). LC-MS/MS analyses were then carried out at our institutional MS facility, as described (21). Proteins thusly identified were sorted by the fold change scores FC‐A or FC‐B. The original mass spectrometry proteomics data have been deposited to the ProteomeXchange Consortium *via* the PRIDE (www.ebi.ac.uk/pride/) partner repository (22) with the dataset identifier PXD027055.

### Statistical Analyses

All data are represented as mean ± SEM. Unless otherwise stated, statistical differences were analyzed by Student’s t test for paired data, using Prism 9 software (GraphPad, San Diego, CA, USA).

## Results

### Identification of Cellular Processes Downstream of Kinases Controlling the Early Phase of NET Formation

**Figure 1:**
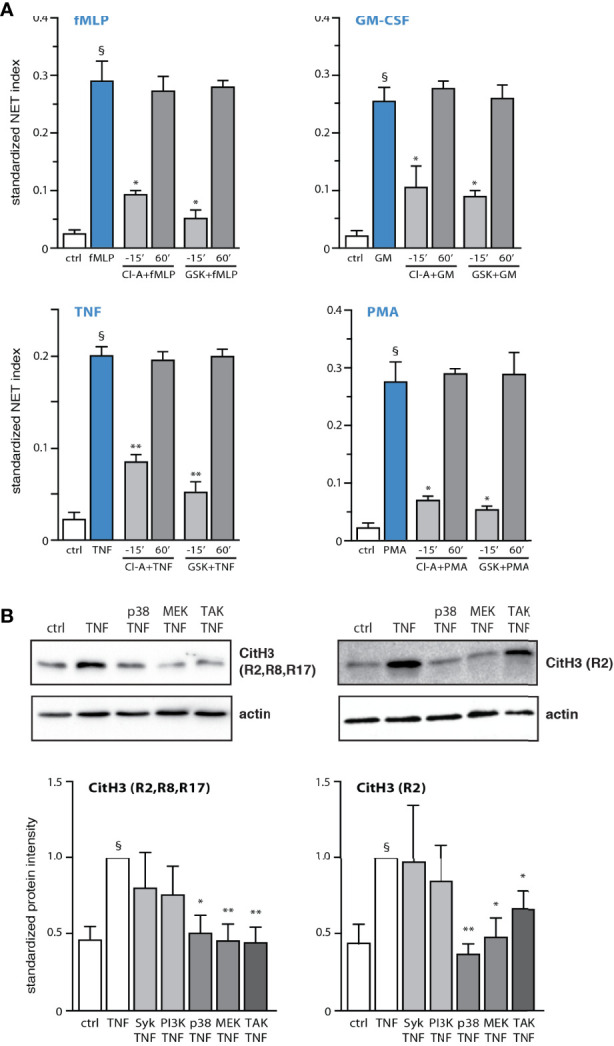
PAD4 activation and histone citrullination are early events in NET generation and are controlled by kinases affecting early processes. **(A)** Neutrophils cultured on poly-L-lysine-coated coverslips were treated either before or after stimulation for the indicated times with 10 µM chloraminidine (“Cl-A”, a general PAD inhibitor), 10 µM GSK484 (a PAD4 inhibitor), or their diluent (DMSO). The cells were stimulated for 4h in the absence (“ctrl”) or presence of 30 nM fMLP, 1 nM GM-CSF, 100 U/ml TNFα, or 50 nM PMA. NET formation was then assessed using PlaNET Blue, as described in *Methods*. Quantitative representation of these experiments, expressed as NET index. Mean ± s.e.m. from at least 3 independent experiments. *p < 0.05; **p < 0.01 *vs* stimulus alone. ^§^p < 0.015 *vs* unstimulated cells. **(B)** Neutrophils cultured on poly-L-lysine-coated coverslips were pre-treated (15 min) with the following inhibitors or their diluent (DMSO): 10 µM piceatannol (Syk inhibitor); 10 µM LY294002 (PI3K inhibitor); 1 µM SB202190 (p38 MAPK inhibitor); 10 µM U0126 (MEK inhibitor); 1 µM (5Z)-7-oxozeaenol (TAK1 inhibitor). The cells were then further incubated for 10 min in the absence (“ctrl”) or presence of 100 U/ml TNFα. Samples were then processed for immunoblot detection of citrullin residues on histone H3, as depicted. A representative experiment is shown, along with a quantitative compilation of these experiments (mean ± s.e.m. from at least 4 independent experiments; *p < 0.05 *vs* stimulus alone; **p < 0.01 *vs* stimulus alone; ^§^p < 0.005 *vs* unstimulated cells).

Having recently established that discrete signaling cascades control early and late events driving NET formation in response to several physiological stimuli (13, 14), we sought to identify some of these events. We initially focused on histone citrullination as a potential early process, as histone H3 citrullination reportedly occurs within 30 min in neutrophils exposed to LPS, TNFα, or fMLP (13, 23), and in view of our finding that PAD4 is required for both this process and for NET formation (13, 14). As shown in **Figure 1A**, the addition of PAD inhibitors before neutrophil stimulation with physiological agonists or PMA largely prevents NET production, as previously reported (13, 14), whereas addition of the inhibitors after the stimulus is ineffective, even after only 30 min (our unpublished data). Thus, PAD4 activation is an early event in NET formation, for all stimuli tested. Because this results in the citrullination of histones (among known substrates), we next monitored this very process, by taking care to incubate the cells exactly as we do when conducting NET assays (i.e. neutrophils adhering to poly-L-lysine-coated coverslips). Time course experiments showed that histone H3 citrullination is detectable as early as 5 min after neutrophil stimulation with TNFα or fMLP (**Figure S1**), confirming that this is an early event. Further investigation revealed that the citrullination of histone H3 occurred mostly on residue R2 (**Figure 1B**). By contrast, the citrullination of residues R8 and R17 of histone H3, or of histone H4 on residue R3, was either weak or undetectable, regardless of the neutrophil activation state (not shown). We also determined which signaling components act upstream of histone citrullination. As shown in **Figure 1B**, neutrophil pretreatment with inhibitors of TAK1, MEK, or p38 MAPK markedly attenuated the citrullination of histone H3, whereas this response was unaffected following inhibition of kinases controlling late stages of NET generation (e.g. Syk, PI3K). Thus, PAD4 activation and histone citrullination are immediate-early events occurring downstream of signaling pathways that control the early phase of NET production.

### Identification of Cellular Processes Involved in the Late Phase of NET Formation

**Figure 2:**
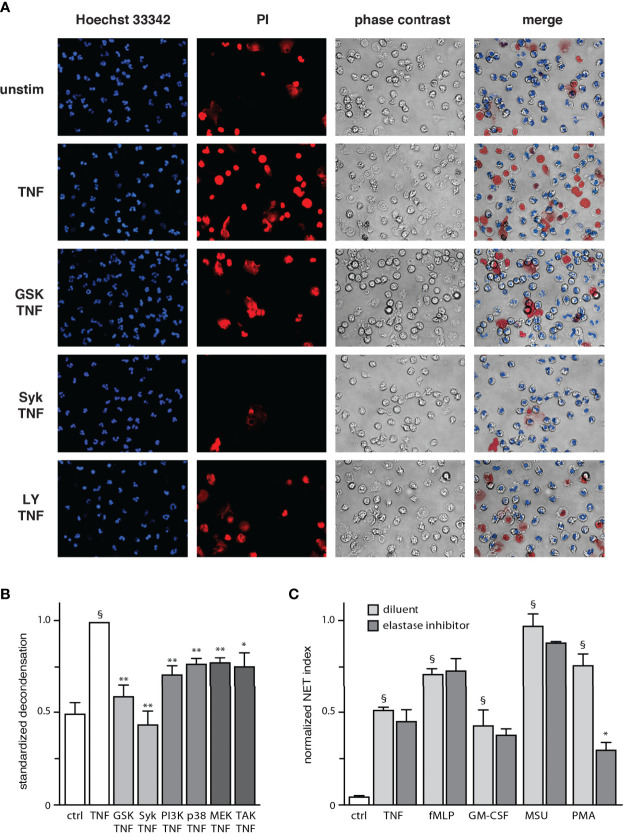
Chromatin decondensation is a late event in NET generation. **(A)** Neutrophils cultured on poly-L-lysine-coated coverslips in a culture incubator were pre-treated (15 min) with the following inhibitors or their diluent (DMSO): 10 µM piceatannol (Syk inhibitor); 10 µM LY294002 (PI3K inhibitor); or 10 µM GSK484 (PAD4 inhibitor). The cells were then incubated at 37°C for 2.5 h in the absence or presence of 100 U/ml TNFα, at which point the following nuclear dyes were added: Hoechst 33342 (cell-permeable, to stain all nuclei) and propidium iodide (cell-impermeable, to stain cells whose membrane had ruptured). The cells were then further incubated at 37°C for another 30 min (3.5 h total stimulation time) before confocal microscope analysis. A representative experiment is shown. **(B)** Quantitative compilation of these experiments, for which the % cells with nuclei showing decondensed chromatin was standardized to the value of stimulated cells. Mean ± s.e.m. from at least 3 independent experiments; *p < 0.04 *vs* stimulus alone; **p < 0.01 *vs* stimulus alone; §p < 0.001 *vs* unstimulated cells). **(C)** Neutrophils cultured on poly-L-lysine-coated coverslips were treated 15 min before stimulation with 10 µM GW311616A (an elastase inhibitor) or its diluent (DMSO). The cells were stimulated for 4 h in the absence (“ctrl”) or presence of 30 nM fMLP, 1 nM GM-CSF, 100 U/ml TNFα, 1 mg/ml MSU, or 50 nM PMA. NET formation was then assessed using PlaNET Blue, as described in *Methods*. Quantitative representation of these experiments, expressed as NET index. Mean ± s.e.m. from 3 independent experiments. *p < 0.05 *vs* stimulus alone. ^§^p < 0.014 *vs* unstimulated cells.

Although a small extent of chromatin extrusion becomes detectable by 90 min of stimulation in our system, a robust response typically requires about 4 h (**Figure S2**). Accordingly, our initial work revealed that a substantial number of neutrophils displaying decondensed chromatin can be observed about one hour before large-scale extrusion (not shown). Thus, chromatin decondensation must represent a late process in NET formation. To investigate this possibility, we carried out live-cell experiments in which stimulated neutrophils were cultured at 37°C on poly-L-lysine-coated coverslips for 2.5 h, at which point we added a cell-permeable nuclear dye (to track changes in chromatin compaction) as well as propidium iodide (to allow for a visualization of intracellular DNA following cell membrane rupture). Subsequent time-lapse confocal microscopy analysis showed that while most TNF-stimulated neutrophils assumed a normal morphology for the first 3 h or so, chromatin decondensation became increasingly frequent over the next hour (as evidenced by nuclear swelling), culminating in propidium iodide entry (**Movie S1**). Under these experimental conditions, chromatin extrusion was mostly undetected since incubation of neutrophils in the presence of cell-permeable DNA dyes largely prevents this response (**Figure S3**), thereby enabling the observation of cells featuring round, swollen nuclei. As shown in **Figure 2A**, decondensed chromatin was indeed evident in TNF-stimulated cells after 3.5 hours; a similar outcome was observed using fMLP as a stimulus (not shown). We also examined which signaling pathways affect chromatin decondensation. As shown in **Figure 2B**, all inhibitors used hindered the phenomenon. Inhibition of Syk and PAD4 proved to be particularly potent, while other inhibitors brought decondensation levels about half-way back to those observed in unstimulated cells (**Figure 2B**). Differences in potency between inhibitors were not found to be statistically significant by one-way ANOVA analysis. Thus, chromatin decondensation is a late event in NET formation, that is affected by kinases controlling both the early and late phases of the phenomenon. Because elastase has been proposed to play a role in initiating decondensation in PMA-activated neutrophils (3), we also investigated whether this mechanism might be involved. However, we found that elastase inhibition does not affect NET formation in response to various physiological stimuli (**Figure 2C**); only PMA-induced NET formation was affected, as previously reported (3, 24).

### Neutrophils Condition Themselves to Become Poised for NET Induction

**Figure 3:**
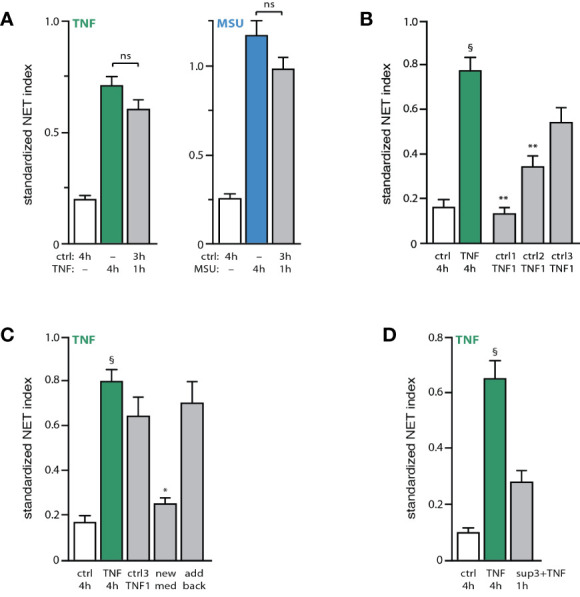
Unstimulated neutrophils condition themselves to quickly form NETs upon stimulation. **(A)** Neutrophils cultured on poly-L-lysine-coated coverslips were incubated for 4 h in medium alone (“ctrl”) or with either 100 U/ml TNFα or 1 mg/ml MSU. Alternatively, cells were incubated 3 hours in medium alone and the stimulus was added for another hour. NET formation was assessed using PlaNET Green (TNF) or PlaNET Blue (MSU) as described in *Methods*. Mean ± s.e.m. from 4 independent experiments. Cell stimulation with TNF or MSU alone yielded significant (p<0.001) differences *versus* unstimulated cells. **(B)** Neutrophils cultured as described above were incubated for 1, 2, or 3 h in medium alone (“ctrl1, ctrl2, ctrl3”), prior to a 1-h stimulation with 100 U/ml TNFα (“TNF1”). As a control, cells were also incubated for 4 h in the absence (“ctrl”) or presence of TNFα. NET formation was assessed using PlaNET Green as described in *Methods*. Mean ± s.e.m. from 3 independent experiments. **p < 0.003 *vs* TNF 4h; ^§^p < 0.002 *vs* unstimulated cells. **(C)** Neutrophils were cultured for 4 h in the absence (“ctrl”) or presence of 100 U/ml TNFα (first two bars). Alternatively, cells were incubated 3 h in medium alone, at which point one of three procedures were followed: 100 U/ml TNFα was added and the cells were further incubated for 1 h (“ctrl3 TNF1”); or the cultured supernatant was removed and replaced with fresh medium (supplemented with 2% autologous serum) and the cells further incubated for 1 h in the presence of exogenous TNFα (“new med”); or the cultured supernatant was removed, added back, and the cells were further incubated for 1 h in the presence of exogenous TNFα (“add back”). NET formation was assessed using PlaNET Green as described in *Methods*. Mean ± s.e.m. from 3 independent experiments. *p < 0.02 *vs* TNF 4h; ^§^p < 0.002 *vs* unstimulated cells. **(D)** Neutrophils were cultured for 4 h in the absence (“ctrl”) or presence of 100 U/ml TNFα (first two bars). Alternatively, cells were incubated for 1 h in the presence of both exogenous TNFα (100 U/ml) and supernatants from unstimulated neutrophils that had been cultured for 3h (“sup3+TNF”). NET formation was then assessed using PlaNET Green as described in *Methods*. Mean ± s.e.m. from 3 independent experiments. ^§^p < 0.02 *vs* unstimulated cells.

Because the first observable sign of impending NET production (i.e. chromatin decondensation) occurs some 3 h following stimulation under our experimental conditions, we examined whether neutrophils might condition themselves to extrude chromatin in response to a stimulus. To explore this possibility, unstimulated neutrophils were incubated for 3 h, and a strong physiological stimulus (TNF or MSU) was then added for another hour. As shown in **Figure 3A**, this resulted in a robust NET formation that did not differ significantly from the one observed when the stimulus alone was added continuously for 4 h. Thus, unstimulated neutrophils seem to condition themselves for rapid NET formation. To determine whether this conditioning requires 3 h to occur, unstimulated neutrophils were next incubated for increasing lengths of time, prior to stimulus addition for another hour. As shown in **Figure 3B**, a one-hour pre-incubation was insufficient to allow neutrophils to quickly generate NETs in response to TNFα; however, a two-hour pre-incubation made it possible to detect some NET formation, and a three-hour pre-incubation allowed for a large-scale response that did not significantly differ from that resulting from a 4-h stimulation with TNFα. Thus, neutrophils become poised for NET induction in what appears to be yet another late process. To gain further insight into this phenomenon, we investigated whether the added stimulus alone was sufficient to trigger rapid NET formation in neutrophils incubated for 3 h, or whether neutrophils also release factors during the first 3 h of incubation, which are required in addition to the subsequently added stimulus. As shown in **Figure 3C**, the presence of the culture supernatant prior to the late addition of TNFα was essential for rapid NET formation, as its replacement with fresh culture medium prevented the response from taking place. This lack of effect could not be attributed to the removal of the supernatant from the unstimulated cells, since adding back the same supernatant along with exogenous TNF led to a robust NET formation within 1 h (**Figure 3C**, last bar). These observations indicate that endogenous factors released by unstimulated neutrophils are necessary to elicit a rapid NET formation in response to a *bona fide* stimulus. This also prompted us to examine whether exposing naïve neutrophils to both an exogenous stimulus and supernatants from 3h unstimulated neutrophils would result in rapid NET formation (i.e. within 1h). As shown in **Figure 3D**, this only resulted in a weak NET response, which did not differ significantly from baseline. Thus, unstimulated neutrophils do not only release factors that act along with a subsequently added stimulus to quickly entail NET formation; the cells must additionally be conditioned by these endogenous factors.

### Activated Neutrophils Release Endogenous Factors That Promote NET Induction

**Figure 4:**
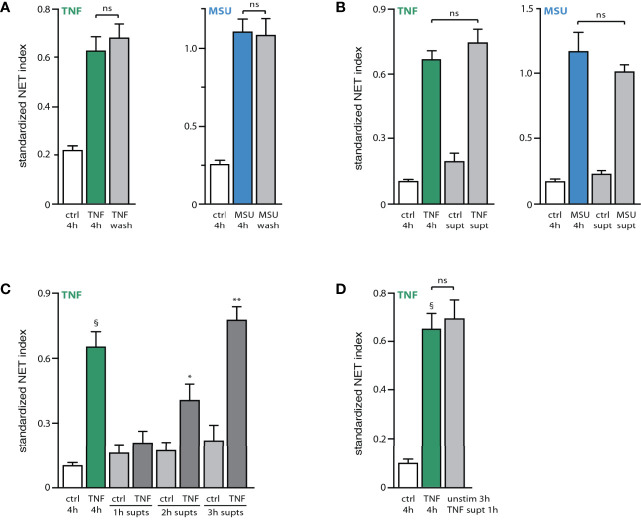
Stimulated neutrophils release endogenous NET-inducing factors. **(A)** Neutrophils cultured on poly-L-lysine-coated coverslips were incubated for 4 h in medium alone (“ctrl”) or with either 100 U/ml TNFα or 1 mg/ml MSU. Alternatively, cells were incubated 15 min with the stimulus, after which time culture supernatants were removed, the cells washed with culture medium, and further incubated with fresh culture medium for the remainder of a total 4-h incubation (“wash”). NET formation was assessed using PlaNET Green (TNF) or PlaNET Blue (MSU) as described in *Methods*. Mean ± s.e.m. from 4 independent experiments. Cell stimulation with TNF or MSU (with or without stimulus removal after 15 min) yielded significant (p ≤ 0.003) differences *versus* unstimulated cells. **(B)** Neutrophils cultured as described above were incubated for 3 h in the absence or presence of a stimulus (100 U/ml TNFα or 1 mg/ml MSU). The culture supernatants were collected and immunodepleted of TNFα, or depleted of MSU crystals by centrifugation, as described in *Methods*. The resulting supernatants (“ctrl supt”, “TNF supt” or “MSU supt”) were then added to naïve neutrophils and incubated for 4 h. For comparison, cells were also incubated for 4 h in the absence (“ctrl”) or presence of TNFα or MSU. NET formation was assessed using PlaNET Green (TNF) or PlaNET Blue (MSU) as described in *Methods*. Mean ± s.e.m. from at least 3 independent experiments. Cell stimulation with TNF or MSU yielded significant (p < 0.023) differences *versus* unstimulated cells. **(C)** Neutrophils cultured as described above were incubated for up to 3 h in the absence or presence of 100 U/ml TNFα. The culture supernatants were collected and immunodepleted of TNFα as described in *Methods*, prior to being added to naïve neutrophils and incubated for 4 h. For comparison, cells were also incubated for 4 h in the absence (“ctrl”) or presence of TNFα or MSU (first two bars). NET formation was assessed using PlaNET Green as described in *Methods*. Mean ± s.e.m. from 3 independent experiments. §p < 0.02 *vs* 4h unstimulated cells; *p < 0.04 *vs* 4h unstimulated cells; **p < 0.01 *vs* 4h unstimulated cells. **(D)** Neutrophils were cultured for 4 h in the absence (“ctrl”) or presence of 100 U/ml TNFα. Alternatively, cells were incubated for 3 h in the presence of TNFα; supernatants were collected, immunodepleted of TNFα, and added for 1 h to unstimulated neutrophils that had been already cultured for 3 h (“unstim 3h + TNF supt 1h”). NET formation was assessed using PlaNET Green as described in *Methods*. Mean ± s.e.m. from 3 independent experiments. ^§^p < 0.02 *vs* unstimulated cells. ns, not significant.

The existence of early and late signaling events and processes in NET formation prompted us to determine whether the continued presence of a stimulus is needed to elicit both early and late events. For this purpose, neutrophils were either stimulated for 4 h, or exposed to the stimulus for 15 min, washed, and further incubated in fresh culture medium for the remainder of the 4-h experiment. As shown in **Figure 4A**, stimulus removal after 15 min had no effect on NET formation, showing that initial exposure is enough to trigger all the needed cellular processes. The occurrence of the phenomenon without the continued presence of the stimulus raised the possibility, that neutrophils might release factors triggering NET formation at later incubation times. To test this hypothesis, supernatants were collected from TNF- or MSU-activated neutrophils for 3 h (i.e. before NET formation, to avoid the release of intracellular contents resulting from plasma membrane rupture). These supernatants were then depleted from their stimulus, and co-incubated with unstimulated neutrophils for 4 h. As shown in **Figure 4B**, this resulted in a robust NET formation that was akin to that achieved by the matching exogenous stimulus. This was not due to the presence of residual initial stimulus in the supernatants from activated neutrophils, since a second round of TNF immunodepletion (or a second 18,000 g centrifugation in the case of MSU-treated neutrophils) failed to alter the NET-inducing properties of the supernatants (not shown). Likewise, a potential endotoxin contamination of the Sepharose beads used to deplete TNF-Ab complexes is not likely to account for the effect of these stimulus-depleted supernatants, since it would require a staggering amount of contamination, and because no such beads were used in the case of MSU supernatants. Finally, the NET-inducing properties of the stimulus-depleted supernatants cannot be attributed to the presence of extracellular vesicles, as the latter are pelleted when these supernatants are spun at 18,000 g, prior to their addition to fresh neutrophils.

In contrast to supernatants from stimulated neutrophils, those collected from unstimulated cells consistently failed to elicit NET formation (**Figure 4B**). Some endogenous factors are therefore released by activated neutrophils, which differ from the initial stimulus, and which promote NET formation. This seems to represent a late event, as 1-h supernatants from activated neutrophils contained no such endogenous factors, whereas their presence became significant in 2-h supernatants from activated cells, and a full-scale effect was achieved using 3-h supernatants (**Figure 4C**). As in the case of exogenous stimuli, stimulus-depleted culture supernatants from activated neutrophils (3h) could trigger NET formation within 1 h when added to unstimulated neutrophils that had been in culture for 3 h (**Figure 4D**). Thus, supernatants from activated neutrophils contain an endogenous stimulatory activity that acts much like an exogenous stimulus of NET formation.

**Figure 5:**
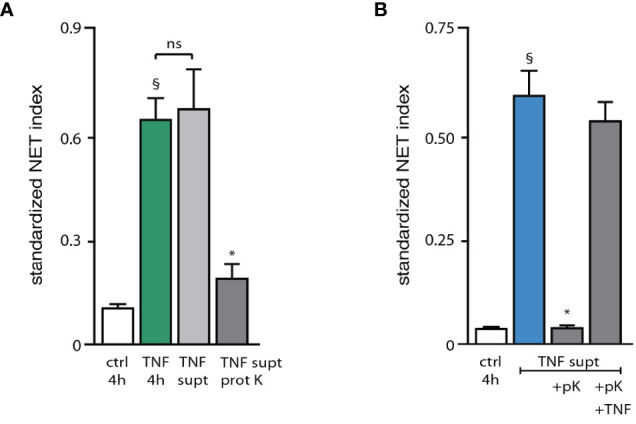
Characterization of the endogenous NET-inducing factors released by activated neutrophils. **(A)** Neutrophils cultured on poly-L-lysine-coated coverslips were incubated for 4 h with medium alone (“ctrl”) or with 100 U/ml TNFα. Alternatively, cells were incubated for 3 h in the presence of TNFα. Supernatants were collected, immunodepleted of TNFα as described in *Methods*, and incubated for 3 h at 37°C in the absence or presence of proteinase K, prior to being added to freshly cultured neutrophils for 4 h (“TNF supt” and “TNF supt prot K”, respectively). NET formation was assessed using PlaNET Green as described in *Methods*. Mean ± s.e.m. from 3 independent experiments. ^§^p < 0.02 *vs* unstimulated cells; *p < 0.04 *vs* TNF supernatants without proteinase K. **(B)** Cells were cultured as described above and incubated for 4 h with medium alone (“ctrl”). Alternatively, cells were stimulated for 3 h with 100 U/ml TNFα. Supernatants were collected, immunodepleted of TNFα, and incubated for 3 h at 37°C in the absence or presence of proteinase K, prior to being added to freshly cultured neutrophils for 4 h (“TNF supt” and “TNF supt +pK”, respectively). In the latter instance, cells cultured for 4 h with proteinase K-digested supernatants were also exposed to 100 U/ml exogenous TNFα (“TNF supt +pK +TNF”). NET formation was assessed using PlaNET Blue as described in *Methods*. Mean ± s.e.m. from 3 independent experiments. §p = 0.01 *vs* unstimulated cells; *p = 0.01 *vs* TNF supernatants alone (2nd bar). ns, not significant.

To gain further insight into the nature of this endogenous stimulatory activity, stimulus-depleted culture supernatants from activated neutrophils were digested with proteinase K prior to their addition to fresh neutrophils for 4 h. As shown in **Figure 5A**, this largely eliminated the NET-inducing properties of these supernatants, indicating that the main factor(s) involved are peptides or proteins. In control experiments, TNFα was added back following proteinase K digestion of supernatants from activated neutrophils, resulting in a NET generation similar to that achieved using only exogenous TNFα as stimulus (**Figure 5B**). This confirms that proteinase K was properly inactivated after its digestion of the supernatants from activated neutrophils, and that its presence did not interfere with the ability of neutrophils to form NETs in response to an exogenous stimulus. We next sought to determine which proteic factors were present in culture supernatants from activated neutrophils, relative to the supernatants from unstimulated cells. To this end, supernatants from neutrophils cultured in the absence of serum (which retain the ability to form NETs) were immunodepleted of the stimulus (TNFα), tested for their ability to induce NET formation (**Figure S4**), and processed for mass spectrometry proteomics analysis. This was done in two independent experiments, each performed using neutrophils from different blood donors. Some 1800 proteins with a minimum of 1 unique peptide match were identified in this manner; this could be narrowed down to some 640 proteins featuring 2 unique peptide matches or more. Among them, fewer than 150 proteins were induced at least 2-fold in culture supernatants harvested from activated neutrophils, relative to those of unstimulated cells (**Table S1**). **Table 1** shows the 34 upregulated proteins that were common to both mass spectrometry experiments, grouped by cellular function.

**Table 1:** Common proteins featuring featuring at least a 2-fold induction (TNF *vs* unstimulated), in two independent experiments.

gene name	protein name	fold induction		known cellular function
		**expt 1**	**expt 2**	
ARPC2	Actin Related Protein 2/3 Complex Subunit 2	6.89	7.24	Cytoskeleton Related (control of actin polymerization)
ARPC3	Actin Related Protein 2/3 Complex Subunit 3	3.26	3.34	Cytoskeleton Related (control of actin polymerization)
ARPC4-TTLL3	Actin Related Protein 2/3 Complex Subunit 4	3.26	4.12	Cytoskeleton Related (control of actin polymerization)
D6PXK4	Alpha Actinin-4	6.89	3.34	Cytoskeleton Related (control of actin polymerization)
F6USW4	F-actin capping protein subunit beta	6.89	4.12	Cytoskeleton Related
FLNA	Filamin A	3.87	7.91	Cytoskeleton Related (actin-binding protein)
IQGAP1	Ras GTPase-activating-like protein	4.17	2.56	Cytoskeleton Related (scaffold protein for actin cytoskeleton)
RAB7A	Rab-7a	3.26	4.90	Cytoskeleton Related (microtubule-directed endosomal migration)
TLN1	Talin-1	5.08	3.34	Cytoskeleton Related
TUBB3	Tubulin Beta 3 Class III	2.36	4.12	Cytoskeleton Related
VASP	Vasodilator-stimulated phosphoprotein	3.26	4.90	Cytoskeleton Related (actin-associated cytoskeleton remodeling)
VCL	Vinculin	7.79	4.12	Cytoskeleton Related (links integrins to actin cytoskeleton)
ADSS	Adenylosuccinate Synthase	3.72	3.34	Metabolism
E9PMM6	Glycogen phosphorylase	14.58	11.93	Metabolism
G6PD	Glucose-6-Phosphate Dehydrogenase	4.12	6.46	Metabolism
HK3	Hexokinase 3	8.24	2.56	Metabolism
NME1	Nucleoside diphosphate kinase A	2.81	2.56	Metabolism
Q5SYT8	Nicotinamide phosphoribosyltransferase-like	3.72	4.12	Metabolism
EFHD2	EF-hand domain family member D2	2.81	2.56	Calcium-binding adaptor protein; potential RAGE ligand
GCA	Grancalcin	5.53	5.68	Calcium-binding protein; RAGE ligand
S100A6	S100A6	3.44	2.93	Calcium-binding protein; RAGE ligand
ITGAM	Integrin alpha M precursor	2.46	6.46	Adherence
ITGB2	Integrin Subunit Beta 2 precursor	2.06	3.34	Adherence
ITIH1	Inter-Alpha-Trypsin Inhibitor Heavy Chain 1	5.08	2.42	Protease inhibitor
SERPINB1	Serpin B1	2.73	4.24	Protease inhibitor
B0UZ83	Uncharacterized	2.73	4.12	Other
C4BPA	Complement C4b Binding Protein Alpha chain	2.08	4.50	Other
DDX39B	ATP-Dependent RNA Helicase P47	2.36	4.12	Other
H3F3C	Histone H3.3C	2.75	10.37	Other
HIST1H4L	Histone H4	2.79	2.51	Other
LTA4H	Leukotriene A4 Hydrolase	10.51	3.96	Other
MMP9	MMP-9	4.35	4.90	Other; present on NETs
Q86U12	Uncharacterized; some similarities with HSP-90	5.08	3.34	Other; potential RAGE ligand
GDI2	GDP Dissociation Inhibitor 2	3.22	4.90	Other

In each of the experiments for which we conducted proteomics analysis, we also included a sample of neutrophils left unstimulated for 30 min (**Figure S4**), so that they could be compared with cells left unstimulated for 3h, as the latter contain endogenous factors that allow neutrophils to rapidly generate NETs in response to a stimulus (**Figure 3**). Some 450 proteins with a minimum of 1 unique peptide match were identified in this manner in cells unstimulated for 3h, *versus* some 300 proteins in cells unstimulated for 30 min. As shown in **Table S2**, almost 130 proteins were induced 2-fold or more in supernatants from 3h unstimulated neutrophils, relative to those from cells left unstimulated for 30 min. Among them, about half (64 proteins) were common to both mass spectrometry experiments; they are grouped by cellular function in **Table 2**.

**Table 2:** Common proteins featuring featuring at least a 2-fold induction (neutrophils unstimulated for 3h *vs* for 30 min), in two independent experiments.

gene name	protein name	fold induction		known cellular function
		expt 1	expt 2	
ADSS	Adenylosuccinate synthetase 2	3.77	3.54	Metabolism
CPNE7	Copine 7	2.38	2.27	Metabolism
E9PMM6	Glycogen phosphorylase	7.42	4.56	Metabolism
ENO1	Enolase 1	2.48	3.54	Metabolism
ENOSF1	Enolase Superfamily Member 1	2.38	2.27	Metabolism
G6PD	Glucose-6-Phosphate Dehydrogenase	2.1	3.88	Metabolism
GPI	Glucose-6-Phosphate Isomerase	3.47	9.6	Metabolism
HK3	Hexokinase 3	2.1	3.88	Metabolism
LDHA	Lactate dehydrogenase	3.94	4.85	Metabolism
NME1	Nucleoside diphosphate kinase A	2.85	2.69	Metabolism
PGLS	6-Phosphogluconolactonase	2.85	2.69	Metabolism
PLBD1	Phospholipase B Domain Containing 1	3.31	3.12	Metabolism
Q5SYT8	Nicotinamide phosphoribosyltransferase-like	3.77	3.54	Metabolism
TPI1	Triosephosphate Isomerase 1	2.11	3.5	Metabolism
ACTR2	Actin Related Protein 2	3.96	7.35	Cytoskeleton-related
D6PXK4	Alpha-actinin-4	3.5	6.5	Cytoskeleton-related (actin-binding)
F6USW4	F-actin-capping protein subunit beta	2.33	3.25	Cytoskeleton-related
FLNA	Filamin A	3.94	2.91	Cytoskeleton-related (actin-binding protein)
IQGAP1	Ras GTPase-activating-like protein,	4.23	3.96	Cytoskeleton-related (scaffold protein for actin cytoskeleton)
SPTBN1	Spectrin Beta	2.38	2.27	Cytoskeleton-related (crosslinks actin)
VCL	vinculin	3.96	3.67	Cytoskeleton-related (links integrins to actin)
A6XMW0	Pro-Eosinophil Major Basic Protein	7.46	3.46	MBP is present on eosinophil ETs
BPI	Bactericidal Permeability Increasing Protein	8.39	7.77	Present on NETs
ELANE	Neutrophil elastase	3.77	2.7	Present on NETs
PRG3	Pro Eosinophil Major Basic Protein 2	2.38	2.27	MBP is present on eosinophil ETs
MMP9	MMP-9	2.95	4.1	Present on NETs
GCA	Grancalcin	5.62	5.23	Calcium-binding protein; RAGE ligand
S100A12	S100A12 or calgranulin	2.08	6.4	Calcium-binding protein; RAGE ligand
S100P	S100P	2.81	5.23	Calcium-binding protein; RAGE ligand
HSPA5	Heat shock 70kDa protein	2.38	2.27	Heat shock-related; potential RAGE ligand
HSPA7	Heat shock 70kDa protein 7	3.31	3.12	Heat shock-related; potential RAGE ligand
Q86U12	Uncharacterized; some similarities with HSP-90	5.15	4.81	Heat shock-related; potential RAGE ligand
CSTB	Cystatin B	2.38	2.27	Protease inhibitor
SERPINB10	Serpin B10	7.46	6.92	Protease inhibitor
ARMC8	Armadillo Repeat Containing 8	2.38	2.27	Other
BASP1	Brain Acid Soluble Protein 1	2.38	2.27	Other
CPPED1	Calcineurin-like Phosphoesterase Domain Containing 1	2.85	2.69	Other
CR1	Complement C3b/C4b Receptor 1	2.85	2.69	Other
DDX39B	ATP-Dependent RNA Helicase P47	2.38	2.27	Other
DOT1L	Disruptor of telomeric silencing 1-like	3.31	3.12	Other
EEF1G	Eukaryotic Translation Elongation Factor 1γ	2.38	2.27	Other
FTL	Ferritin Light Chain	2.38	2.27	Other
GCC2	GRIP and coiled-coil domain-containing protein 2	3.77	3.54	Other
GDI2	GDP Dissociation Inhibitor 2	3.27	3.04	Other
H0Y858	Uncharacterized	2.38	2.27	Other
H7BYC5	Uncharacterized	3.31	3.12	Other
HBA2	Hemoglobin A2	2.38	2.27	Other
HBD	Hemoglobin subunit delta	2.85	2.69	Other
IGHD	Immunoglobulin Heavy Constant Delta	2.38	2.27	Other
LSP1	Lymphocyte Specific Protein 1	2.38	2.27	Other
LTA4H	Leukotriene A4 hydrolase	3.56	3.3	Other
MAP2K4	MAP2K4	2.85	2.69	Other
MSH2	MutS Homolog 2	3.77	3.54	Other
NCF1	p47phox	3.77	3.54	Other
OLFM4	Olfactomedin 4	2.58	2.4	Other
PARK7	Parkinsonism Associated Deglycase	2.38	2.27	Other
PEBP1	Phosphatidylethanolamine Binding Protein 1	2.85	2.69	Other
POU4F2	POU Class 4 Homeobox 2	2.38	2.27	Other
PRRC2C	Proline Rich Coiled-Coil 2C	2.38	2.27	Other
RAB5B	Rab5b	2.85	2.69	Other
SH3BGRL	SH3 Domain Binding Glutamate Rich Protein Like	2.85	2.69	Other
TAGLN2	Transgelin 2	2.85	2.69	Other
TSPAN14	Tetraspanin 14	2.38	2.27	Other
U2SURP	U2 SnRNP Associated SURP Domain Containing	2.85	2.69	Other

### RAGE Ligands Represent Important Endogenous Factors Mediating NET Induction

**Figure 6:**
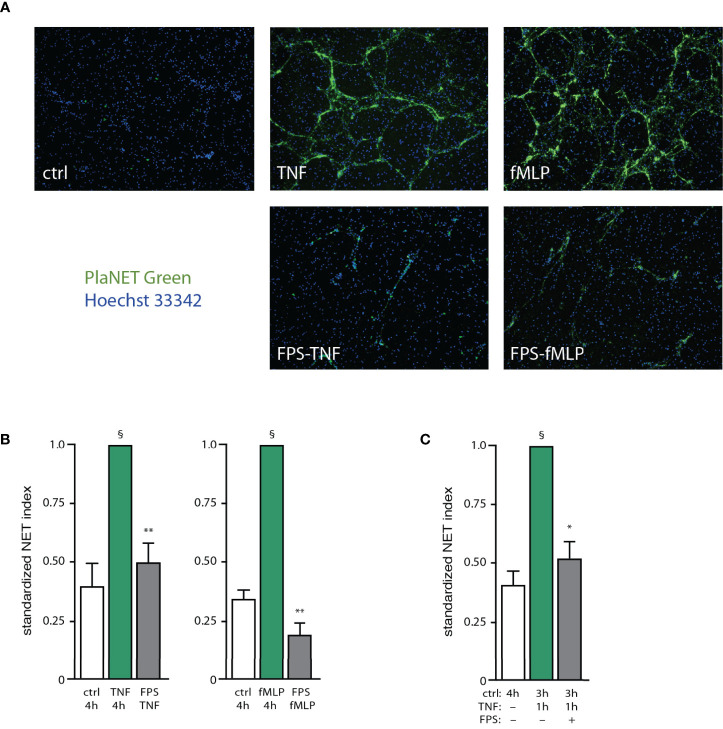
*RAGE ligands are important endogenous factors mediating NET induction*. **(A)** Neutrophils cultured on poly-L-lysine-coated coverslips were incubated for 4 h in medium alone (“ctrl”), or with either 100 U/ml TNFα or 100 nM fMLP. In all cases DMSO was added at the 30-min time point (final concentration, 0.1%). Alternatively, cells were stimulated with either TNF or fMLP for 30 min, then the RAGE antagonist FPS-ZM1 was added (1 µM final concentration, in DMSO), and neutrophils were further incubated for another 3.5 h (“FPS TNF” and “FPS fMLP”, respectively). NET formation was assessed using PlaNET Green as described in *Methods*. A representative experiment is shown (10X magnification). **(B)** Quantitative representation of the above experiments, in which PlaNET Green fluorescence values were standardized according to total cell number (NET index). Mean ± s.e.m. from at least 3 independent experiments. §p < 0.007 *vs* unstimulated cells; **p < 0.003 *vs* stimulus only. **(C)** Neutrophils were cultured for in medium alone (“ctrl”) for the indicated time. In the other conditions (last two bars), cells were incubated 3 h in medium alone, at which point TNFα was added (100 U/ml, final concentration) and the cells were further incubated for 1 h. In one condition (last bar), the cells were exposed to 1 µM FPS-ZM1 after the first 30 min of incubation in medium alone (“FPS”). NET formation was assessed using PlaNET Green as described in *Methods*. Mean ± s.e.m. from 3 independent experiments. *p = 0.021 *vs* TNF only; ^§^p < 0.01 *vs* unstimulated cells.

Several of the potential endogenous NET inducers are *bona fide* or potential RAGE ligands (e.g. S100A proteins, grancalcin, HSP70 analogs, etc). We therefore explored the possibility that such ligands could feed back on neutrophils using this putative common receptor. We first confirmed that S100A9, a RAGE ligand, activates neutrophils as determined by its ability to rapidly promote ERK phosphorylation (**Figure S5A**). We also ascertained that S100A9 elicits NET formation in humans, as reported for mouse neutrophils (25), and that it does so by acting through RAGE since cell pretreatment with FPS-ZM1 (a RAGE antagonist) inhibits the effect of S100A9 (**Figure S5B**). We next investigated whether interfering with RAGE would affect NET formation in response to potent physiological stimuli (e.g. fMLP, TNF). As depicted in **Figures 6A, B**, FPS-ZM1 largely or totally prevented NET generation when added 30 min after either TNF or fMLP; this agrees well with our finding, that endogenous factors contribute to NET formation by acting belatedly. Similar observations were made using other classes of stimuli (e.g. GM-CSF, MSU, PMA), as shown in **Figure S6A**. We could also reproduce these findings using a different approach for NET detection (i.e. Cit H3 visualization), as shown in **Figure S6B**. Finally, we explored whether endogenous RAGE ligands might also contribute to the propension of unstimulated neutrophils to become poised for rapid NET production. To this end, we cultured unstimulated cells for 30 min, added FPS-ZM1 (or its diluent) and incubated the cells for another 2.5 h, prior to stimulation with TNFα for 1 h. As shown in **Figure 6C**, while neutrophils incubated for 3h produced NETs within 1 h of TNF exposure, interfering with RAGE effectively prevented this response. Collectively, these experiments confirm that RAGE ligands rank among the endogenous factors contributing to NET formation in human neutrophils.

## Discussion

The ability of neutrophils to extrude chromatin to entrap invading micro-organisms remains quite fascinating, even some 17 years after its initial discovery. Although some of the underlying mechanisms have been described (e.g. the involvement of PAD isoforms, and under some circumstances the need for endogenous ROS and a nuclear translocation of elastase), our understanding of the phenomenon remains fragmentary. We recently reported that in response to physiological stimuli, NET formation features both early and late events that are controlled by discrete signaling pathways (13, 14). In this study, we identified some of the early and late cellular processes participating in NET formation. In doing so, we unveiled the existence of endogenous factors acting upon neutrophils to mediate NET formation, or to condition the cells to quickly form NETs.

We recently determined that in response to several classes of physiological stimuli, NET formation is largely independent of endogenous ROS, but that conversely, PAD4 involvement is crucial (13, 14). We now show that PAD4 inhibition only affects the early phase (i.e. the first 30 min) of the phenomenon, and that accordingly, the citrullination of histone H3 represents an early neutrophil response that is already detected after 5 min of stimulation. These kinetics confirm and extend previous studies, in which histone H3 deimination was shown to occur within 30 min in response to stimuli such as LPS, TNFα, and fMLP (13, 23). We also went a step beyond, by monitoring the citrullination of individual arginine residues on histone H3, instead of only resorting to the antibody (Abcam ab5103) used by nearly all investigators thus far (including ourselves), which recognizes three citrullinated residues (R2, R8, R17) on histone H3. This approach revealed that contrary to arginine R2, which could be inducibly citrullinated, residues R8 and R17 displayed low levels of citrullination and were not further deiminated in response to neutrophil stimulation. Similar findings were made in the case of histone H4 (R3) citrullination, which was mostly undetectable. Finally, we found that histone H3 citrullination is controlled by the same signaling pathways (TAK1, MEK, p38 MAPK) which we previously showed to affect the initial phase of NET generation (13). Thus, the PAD4-driven citrullination of a discrete residue on H3 represents an early response in neutrophils that will eventually release NETs. While PAD4 undoubtedly participates in NET formation, including in chromatin decondensation (as shown herein), an actual impact of histone citrullination on these processes still awaits a formal demonstration. It has been proposed that histone citrullination somehow favors decondensation since there appears to be a correlation between the intensity of both processes in NETing neutrophils (23, 26), though this could merely reflect the fact that both processes are under the control of PAD4. Similar observations were made in the human osteosarcoma cell line, U2OS, overexpressing PAD4; in this system, H3 citrullination was also observed to cause the dissociation of heterochromatin protein 1β from chromatin, thereby promoting a lesser degree of organization (27). Together, these findings suggest the existence of a link between histone citrullination and decondensation; whether this occurs in neutrophils remains to be established. Alternatively, PAD4 could be driving NET formation by acting through intermediates other than histones, especially since PAD4 has many substrates participating in various cellular responses (28). In this regard, some investigators found that in response to *A. fumigatus*, NET formation is unaffected by PAD4 inhibition, though histone citrullination still is (29). Others reported that NET induction can happen under conditions where no citrullinated H3 is detected (16). Thus, there exist circumstances in which PAD4-driven histone citrullination is uncoupled from NET formation. Further studies are clearly needed to determine whether histone citrullination can contribute to NET formation, or whether it is perhaps a parallel phenomenon.

In contrast to the near-immediate PAD4 activation occurring in response to neutrophil stimulation, chromatin decondensation has been reported to take place after 90 min or more in response to low concentrations of PMA (30, 31). We observed that physiological agonists induce this response in about 3 h in our experimental system, confirming that it indeed represents a late event. More importantly, we show that decondensation is under the control of all the kinases which we previously found to drive NET formation, whether they act early or late (13). This begs the question of which cellular processes are mobilized, that lead to decondensation. In this regard, a role for elastase has been proposed in initiating decondensation, at least in human neutrophils stimulated with PMA (3, 4). Likewise, cathepsin G was shown to act like much like elastase insofar as its binding to DNA promotes histone cleavage (32). However, we found that NET formation in response to various physiological agonists is unaffected by the same elastase inhibitor (GW311616A) that was used by the above investigators, though PMA-induced NET formation was largely abrogated, as per their findings. This might reflect the fact that PMA-triggered NET generation is a NOX-dependent process, whereas physiological agonists elicit this response independently of the oxidase (4, 13, 14, 16, 33, 34). Noteworthy is that neutrophils from elastase-deficient mice generate NETs to a similar extent compared to those from wild-type animals in response to PMA or PAF, and although fewer NETs are made in response to ionomycin, NET formation still takes place (35). Together, these considerations indicate that elastase is probably not an essential component linking signaling events to chromatin decondensation. In support of this conclusion, a recent study found that NET formation occurs independently of elastase and cathepsin G activity (24). Conversely, a recent study showed that PAD4-mediated citrullination allows the calpain-driven proteolysis of proteins bound to the nuclear lamina or chromatin, thereby promoting decondensation of the latter (36). Other investigators additionally reported that gasdermin D, a pore-forming protein, is needed for nuclear expansion and/or chromatin decondensation (37, 38). Thus, a picture of the cellular processes driving decondensation is slowly emerging.

An intriguing finding of the present study is that neutrophils can condition themselves to be poised for subsequent NET induction, and that this represents another late process in NET formation (in addition to chromatin decondensation). We indeed observed that under our experimental conditions, adherent neutrophils cultured for some 3 h in the absence of stimulation acquire the ability to quickly form NETs (within 1 h, as opposed to 4 h) upon exposure to an exogenous stimulus. This behavior requires the presence of the conditioned culture supernatant when the stimulus is added, indicating that neutrophils constitutively release factors that act along with the stimulus to trigger rapid NET formation. Interestingly, the mere addition of conditioned supernatants and exogenous stimuli to naïve neutrophils did not result in the quick generation of NETs. This confirms that neutrophils must condition themselves to endogenous factors, whose continued presence is needed so that they can quickly respond to the exogenous stimulus. As for the nature of these endogenous factors, previous studies (including our own) have shown that neither gene transcription (13, 15, 16) nor protein synthesis (13, 15–17) interferes with NET formation, at least in human neutrophils. Thus, the endogenous factors which condition unstimulated neutrophils must be pre-stored products, as opposed to newly-made proteins. These endogenous factors also differ from the ones present in the supernatants of stimulated neutrophils, as the latter can induce NET formation, whereas supernatants from unstimulated neutrophils do not. This said, it is likely that stimulated neutrophils similarly condition themselves to respond to belatedly produced endogenous NET inducers. In an effort to identify some of the endogenous factors released by unstimulated neutrophils, we compared the proteins present in supernatants from cells that had been cultured for 3 h (which are conditioned for quick NET release), to those of cells cultured for only 30 min (which are not). Our MS proteomics analyses revealed several proteins that were induced 2-fold or more, relative to cells left unstimulated for 30 min (**Table 2**). The most numerous (14) were related to energy metabolism, and included 6 proteins that were further upregulated in TNF-activated neutrophils. Next in abundance were proteins related to the actin cytoskeleton (7), of which 5 were further upregulated in TNF-activated neutrophils, including filamin A, which is reportedly needed for NET formation (39) and α-actinin; in this regard, both proteins were recently shown to be cleaved by calpain following PAD-mediated citrullination (36). Another group of strongly induced proteins consisted of granule constituents that can be found on NETs (BPI, elastase, MMP-9) (40–42), in keeping with the fact that a few NETs can sometimes be observed in unstimulated neutrophils cultured for 3 h, whereas none are ever observed after only 30 min under our experimental conditions. Whether any of these proteins contribute to conditioning the cells for quick NET release, remains to be determined. In this regard, our experiments in which RAGE blockade largely prevented rapid NET induction (**Figure 6C**) have provided some clues, though it still isn’t clear at this juncture whether RAGE ligands participate in the conditioning itself and/or whether they are perhaps needed to act alongside a subsequent stimulus for quick NET generation. Studies are ongoing to elucidate this issue.

In addition to endogenous factors that condition neutrophils to quickly generate NETs, stimulated cells were found to release NET-inducing factors. Both types of endogenous factors are produced within a similar time frame, i.e. in the late stages of NET formation (i.e. at about 2h and beyond). We also provide evidence that the NET-inducing endogenous factors mediate the phenomenon, since it proceeds unaltered when the initial exogenous stimulus is removed after 15 min of exposure (**Figure 4A**). Proteinase K digestion experiments established that the bulk of the endogenous NET-inducing activity was proteic. Mass spectrometry proteomics analyses revealed that between 90 and 140 proteins were increased more than 2-fold in activated neutrophil supernatants, relative to those from unstimulated cells; among them, 34 were common to both experiments. Most of the latter were related to the actin cytoskeleton, or (to a lesser extent) to metabolism, calcium binding, adherence, or antiprotease activity (**Table 1**). It is tempting to speculate that this abundance of actin-related proteins may reflect the cellular changes resulting from (or necessary for) chromatin decondensation and the accompanying nuclear swelling; this might even explain the detection of some histones in the supernatants of activated neutrophils. In the latter instance, extracellular histones have been reported to act as danger-associated molecular patterns that exert pro-inflammatory actions through binding of TLR2/TLR4 (43). It is therefore conceivable that they might activate neutrophils in this manner. Another indication of histones potentially acting as NET-inducing factors is that in contrast to their release by activated neutrophils, they were not upregulated much in supernatants from unstimulated cells incubated for 3 h *versus* those cultured for 30 min. Other common proteins of potential interest that were identified in our MS analyses are MMP-9 (which is present on NETs) (42); and S100A6, which could possibly act as a NET inducer since the related protein, S100A9, induces NET formation in mice (25) and humans (this study). Because S100A proteins and other endogenous factors (e.g. grancalcin, HSP70 analogs) are *bona fide* or potential RAGE ligands, we explored the possibility that one or more could feed back on neutrophils using this common receptor to elicit NET formation. By using a RAGE antagonist, we could confirm this scenario in response to various classes of physiological stimuli (e.g. TNF, fMLP, GM-CSF, MSU) as well as PMA. This suggests that the contribution of secreted endogenous RAGE ligands is a general feature of NET generation. Future studies are needed to identify which endogenous RAGE ligand(s) account for the observed feedback stimulation of neutrophils described herein.

**Figure 7:**
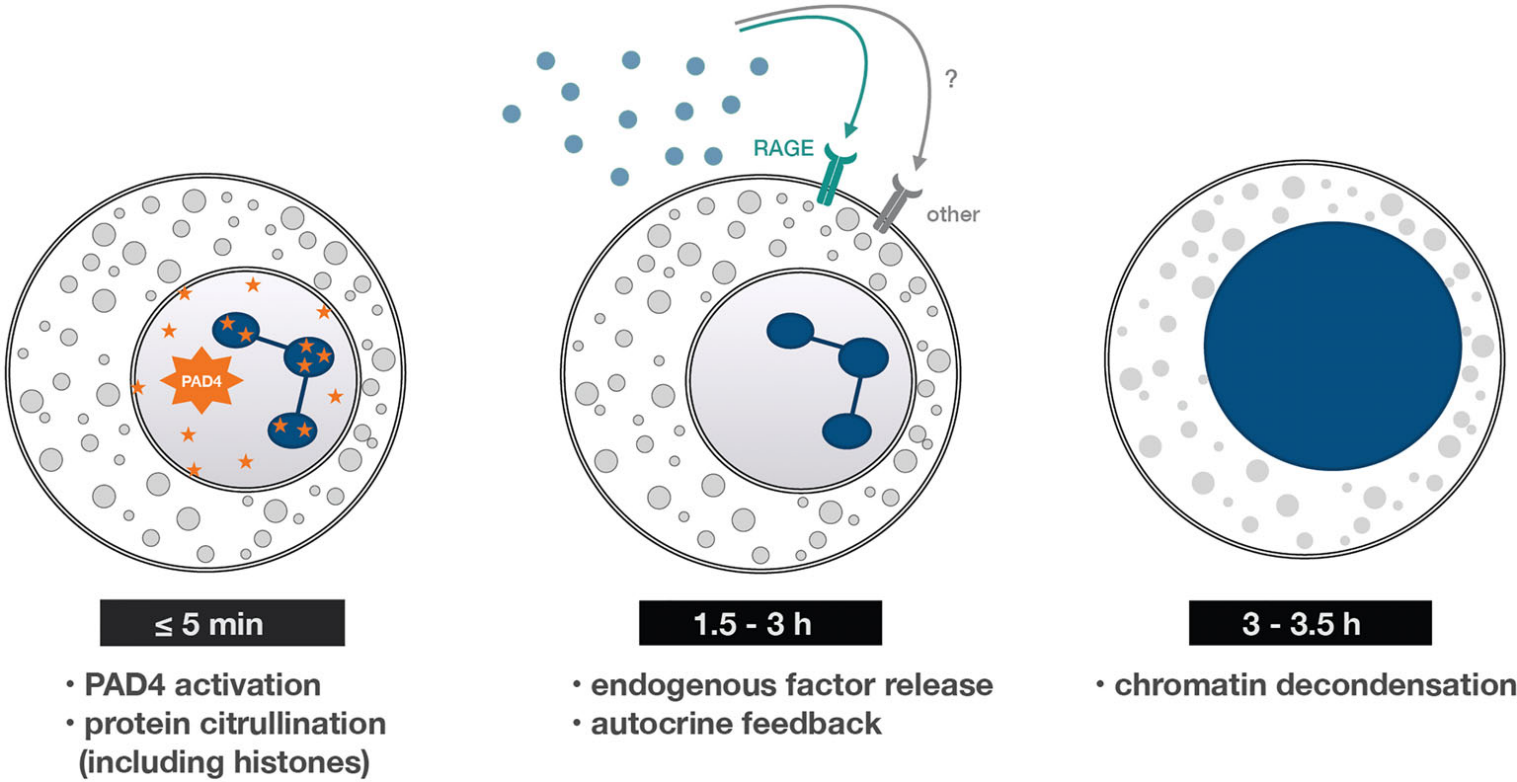
*Early and late processes underlying NET formation*. A summary of the findings reported herein. Within minutes of neutrophil stimulation, PAD4-dependent protein citrullination occurs – an early cellular process needed for NET formation. It then takes some 90-180 min for neutrophils to release RAGE ligands (and perhaps other molecules) that both condition the cells to generate NETs and trigger the later stages of the process. One such late stage is chromatin decondensation, which occurs some 3-3.5 h post-stimulation, and is quickly followed by chromatin extrusion into the extracellular space.

In summary, we deciphered some of the early and late cellular processes underlying NET formation (schematized in **Figure 7**); in particular, we uncovered the existence of endogenous factors that mediate the phenomenon, and that act in an autocrine or paracrine manner through RAGE. This significantly advances our understanding of NET formation and could help spawn new therapeutic strategies, as secreted RAGE ligands represent potential targets for future intervention.

## Data Availability Statement

The original contributions presented in the study are publicly available. This data can be found here: ProteomeXchange *via* the PRIDE repository, with accession number PXD027055.

## Ethics Statement

The studies involving human participants were reviewed and approved by Comité d’éthique de la recherche du CIUSSS de l’Estrie - CHUS. The patients/participants provided their written informed consent to participate in this study.

## Author Contributions

OT carried out the experiments for **Figures 3**–**5**, **S1**, **S4**, **Movie S1**, as well as **Tables 1** and **2**; and provided conceptual input throughout. VC carried out the experiments for **6**, **7**, **S2**, **S5**, **S6B**; most of the experiments in **Figures 1**, **2**; and wrote a part of the *Results* section. HM performed the experiments for **Figures S3** and **S6A**. PM designed the research and mentored the other authors. All authors contributed to the article and approved the submitted version.

## Conflict of Interest

The authors declare that the research was conducted in the absence of any commercial or financial relationships that could be construed as a potential conflict of interest.

## Publisher’s Note

All claims expressed in this article are solely those of the authors and do not necessarily represent those of their affiliated organizations, or those of the publisher, the editors and the reviewers. Any product that may be evaluated in this article, or claim that may be made by its manufacturer, is not guaranteed or endorsed by the publisher.

## Supplementary Material

Supplementary Figure 1 *Kinetics of histone H3 citrullination in human neutrophils*. Cells were cultured on poly-L-lysine-coated coverslips and stimulated with 100 U/ml TNFα **(A)** or 30 nM fMLP **(B)** for the indicated times. Samples were then processed for immunoblot detection of citrullinated histone H3.

Supplementary Figure 2 *Kinetics of NET formation in human neutrophils*. Cells cultured on poly-L-lysine-coated coverslips were stimulated with either 100 U/ml TNFα or 1 mg/ml MSU for the indicated times. NET formation was assessed using PlaNET Green as described in *Methods*. Mean ± s.e.m. from at least 4 representative fields.

Supplementary Figure 3 *Effect of neutrophil co-incubation with cell-permeable DNA dyes on NET formation*. Cells were cultured on poly-L-lysine-coated coverslips for 4 h in the absence (“unstim”) or presence of 100 nM fMLP. In the latter instance, DNA dyes (2 µM Hoechst 33342 or 3 µM DAPI, final concentrations) or their diluent (DMSO, 0.1% final concentration) were added at 3 h post-stimulation. NET formation was assessed using PlaNET Green as described in *Methods*. Mean ± s.e.m. from at least 4 representative fields.

Supplementary Figure 4 *NET-inducing properties of supernatants prior to MS analysis*. Cells were cultured on poly-L-lysine-coated coverslips for 3 h in serum-free RPMI in the absence (“ctrl”) or presence of 100 U/ml TNFα. Supernatants (6 per condition) were collected, pooled, immunodepleted of TNFα as described in *Methods*, and a portion thereof was incubated with freshly isolated neutrophils cultured on poly-L-lysine-coated coverslips for 4 h. NET formation was assessed using PlaNET Green as described in *Methods*.

Supplementary Figure 5 *Effect of exogenous S100A9 on neutrophil responses and involvement of RAGE*. **(A)** Cells (3 x 10^6^/ml) were cultured for 15 min in the absence (“ctrl”) or presence of 20 µg/ml rh S100A9. Samples were then processed for SDS-PAGE analysis and immunoblot detection of P-ERK or β-actin (as a loading control); 10^5^ cell-equivalents were loaded per lane. **(B)** Cells cultured on poly-L-lysine-coated coverslips were incubated in medium alone (“ctrl”), or with 20 µg/ml rh S100A9. At the 30-min time point, the RAGE antagonist FPS-ZM1 (1 µM final concentration) or its diluent (DMSO, 0.1% final concentration) was added, and neutrophils were further incubated for another 3.5 h. NET formation was assessed using PlaNET Green as described in *Methods*. Mean ± s.e.m. from at least 4 representative fields.

Supplementary Figure 6 *Effect of RAGE blockade on NET induction by neutrophil agonists*. **(A)** Cells cultured on poly-L-lysine-coated coverslips were incubated in medium alone (“ctrl”), or with either 1 nM GM-CSF, 1 mg/ml MSU, or 50 nM PMA. At the 30-min time point, the RAGE antagonist FPS-ZM1 (1 µM final concentration) or its diluent (DMSO, 0.1% final concentration) was added, and neutrophils were further incubated for another 3.5 h. NET formation was then assessed using PlaNET Green (or PlaNET Blue in the case of MSU) as described in *Methods*. Mean ± s.e.m. from at least 4 representative fields. **(B)** Neutrophils cultured on poly-L-lysine-coated coverslips were incubated as described above, albeit using 100 nM fMLP as stimulus. NET formation was assessed using Cit H3 antibodies as described before for myeloperoxidase (13).

Movie S1 *Time-lapse video of live neutrophils undergoing chromatin decondensation*. Neutrophils cultured on poly-L-lysine-coated coverslips were stimulated with 100 U/ml TNFα for 2.5 h; Hoechst 33342 and propidium iodide were then added to the culture medium and the cells were placed in the temperature-controlled chamber of a confocal microscope. Pictures were taken every 10 min thereafter, until the total incubation time reached 4h. Magnification is 40X.
