# Supplementary figures and images for: Early and Late Processes Driving NET Formation, and the Autocrine/Paracrine Role of Endogenous RAGE Ligands
Source: Front Immunol. 2021 Sep 20;12:675315. doi: 10.3389/fimmu.2021.675315 (PMC8488397; doi:10.3389/fimmu.2021.675315)

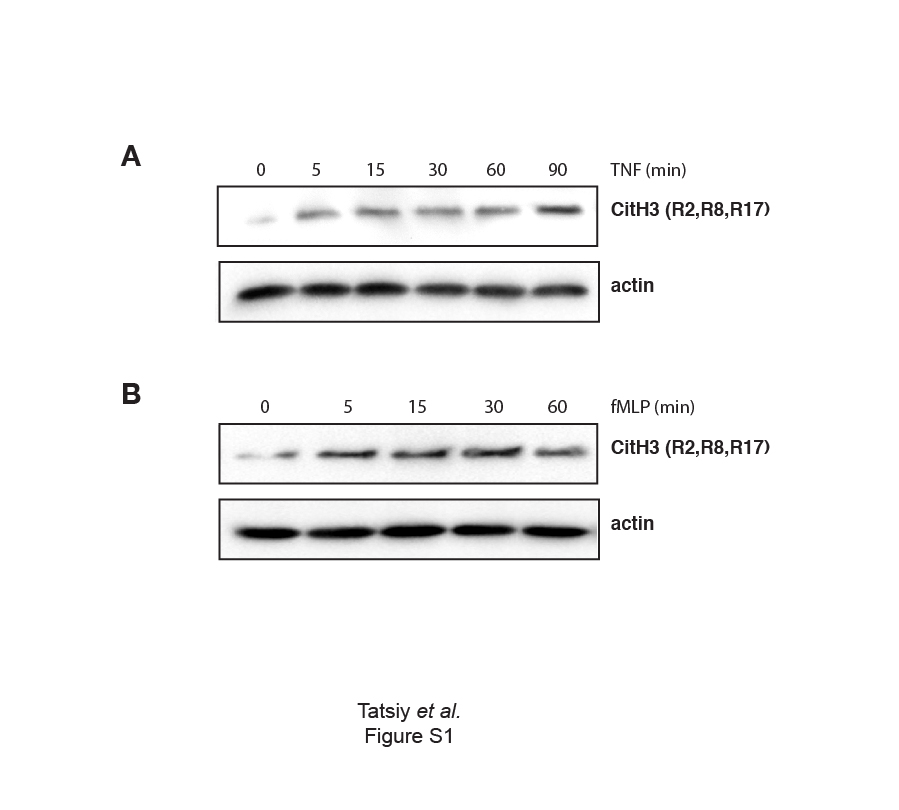

Supplement: Supplementary Figure 1 — Kinetics of histone H3 citrullination in human neutrophils. Cells were cultured on poly-L-lysine-coated coverslips and stimulated with 100 U/ml TNFα (A) or 30 nM fMLP (B) for the indicated times. Samples were then processed for immunoblot detection of citrullinated histone H3. [file Image_1.jpeg]

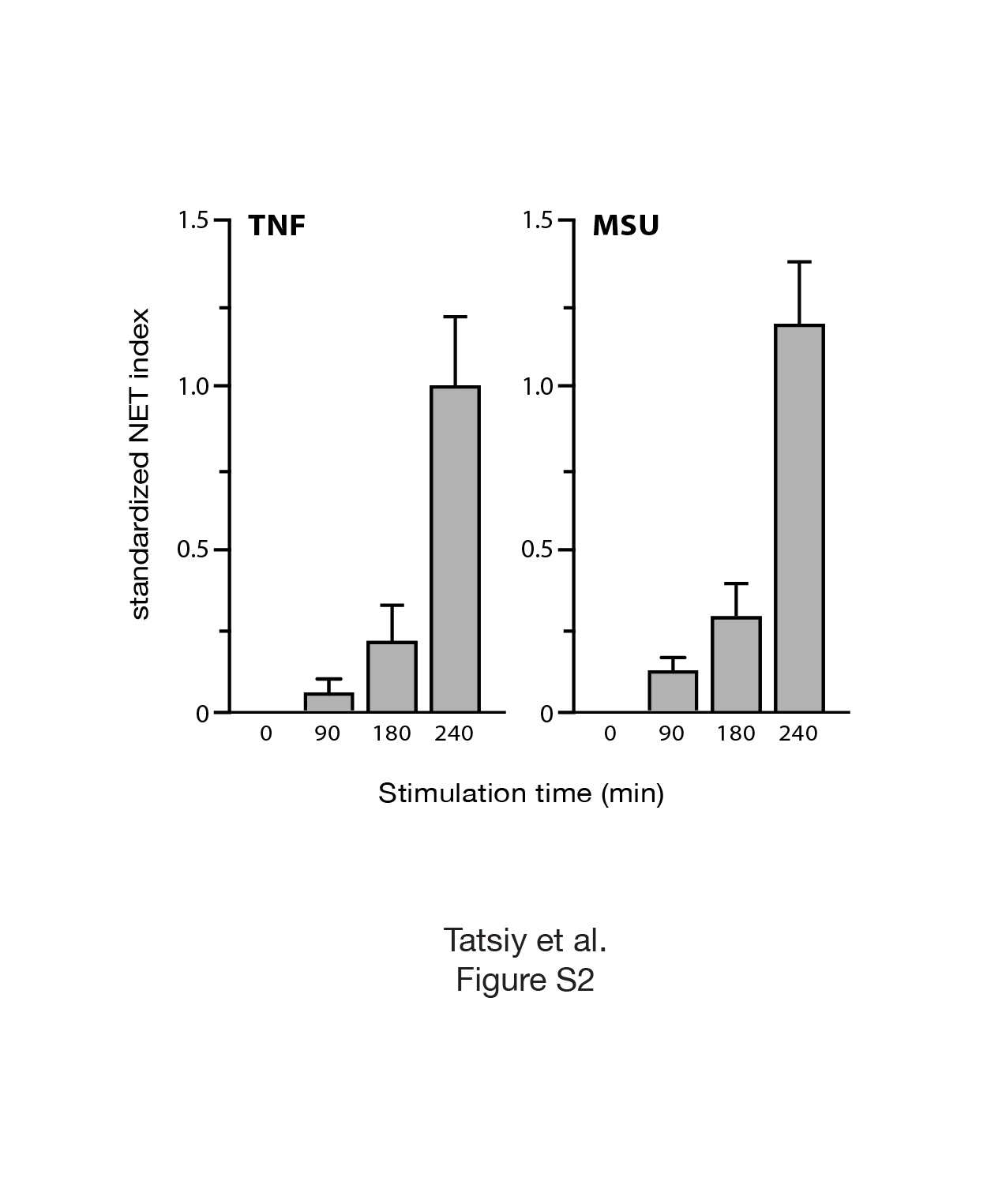

Supplement: Supplementary Figure 2 — Kinetics of NET formation in human neutrophils. Cells cultured on poly-L-lysine-coated coverslips were stimulated with either 100 U/ml TNFα or 1 mg/ml MSU for the indicated times. NET formation was assessed using PlaNET Green as described in Methods. Mean ± s.e.m. from at least 4 representative fields. [file Image_2.jpeg]

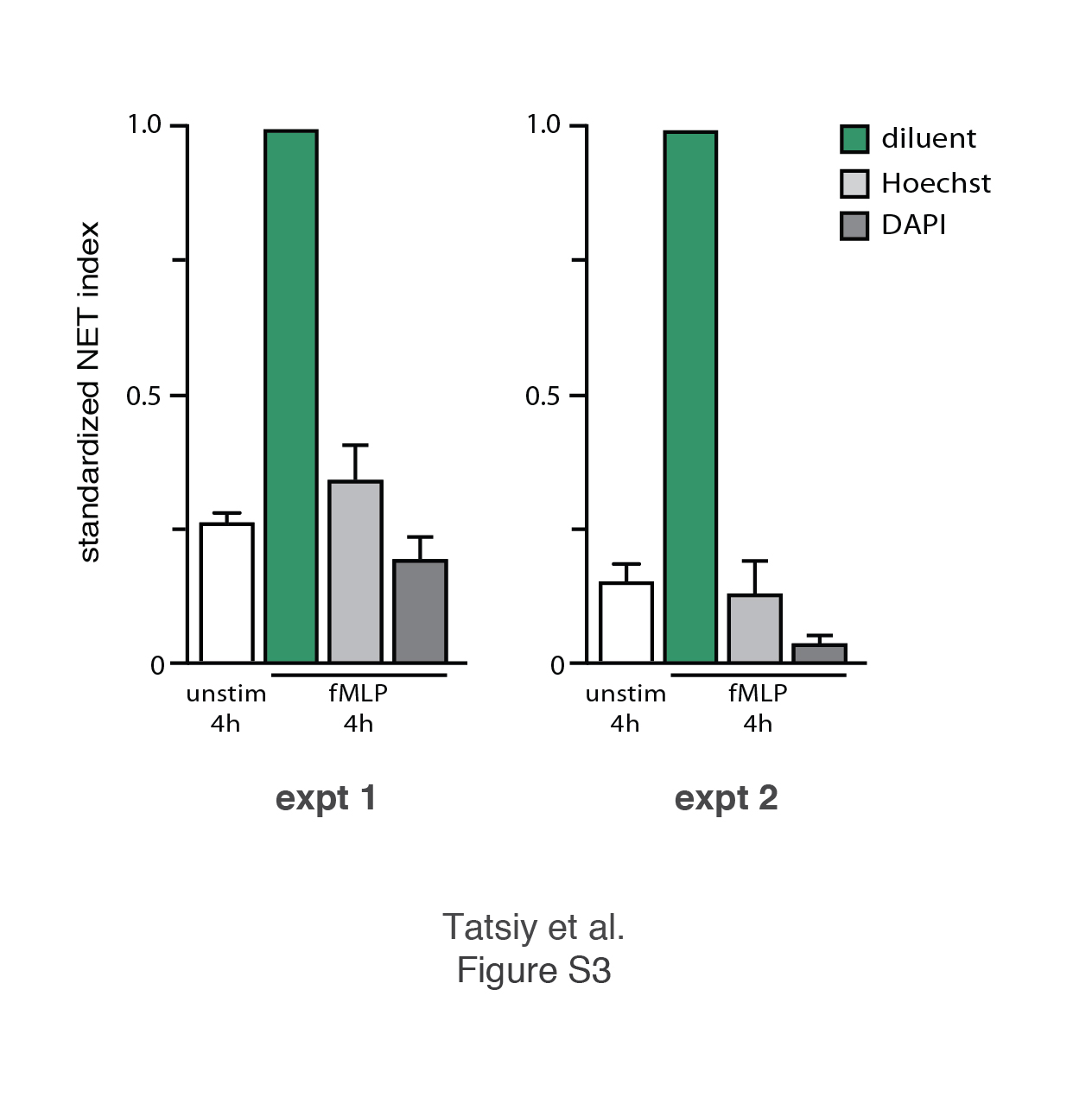

Supplement: Supplementary Figure 3 — Effect of neutrophil co-incubation with cell-permeable DNA dyes on NET formation. Cells were cultured on poly-L-lysine-coated coverslips for 4 h in the absence (“unstim”) or presence of 100 nM fMLP. In the latter instance, DNA dyes (2 µM Hoechst 33342 or 3 µM DAPI, final concentrations) or their diluent (DMSO, 0.1% final concentration) were added at 3 h post-stimulation. NET formation was assessed using PlaNET Green as described in Methods. Mean ± s.e.m. from at least 4 representative fields. [file Image_3.jpg]

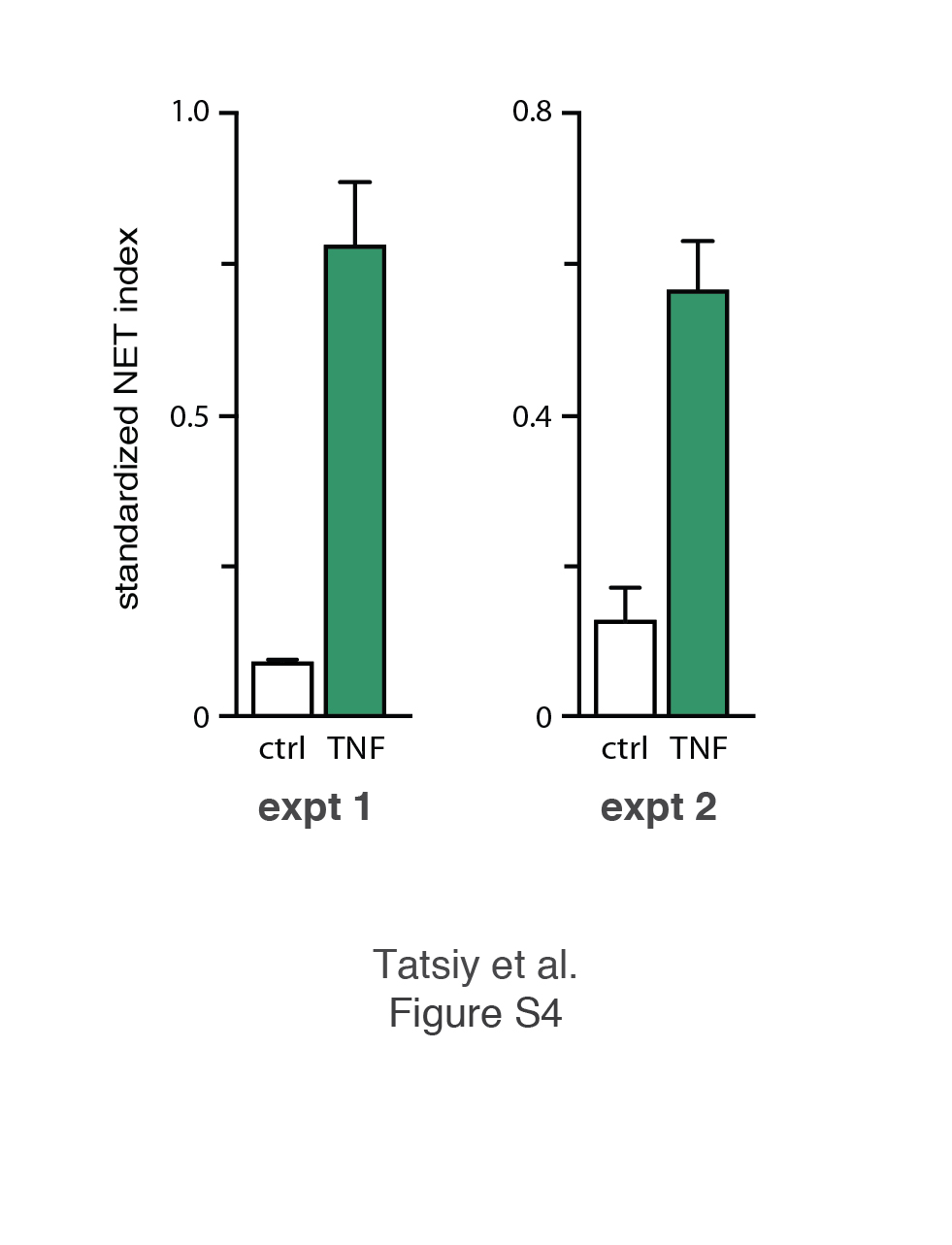

Supplement: Supplementary Figure 4 — NET-inducing properties of supernatants prior to MS analysis. Cells were cultured on poly-L-lysine-coated coverslips for 3 h in serum-free RPMI in the absence (“ctrl”) or presence of 100 U/ml TNFα. Supernatants (6 per condition) were collected, pooled, immunodepleted of TNFα as described in Methods, and a portion thereof was incubated with freshly isolated neutrophils cultured on poly-L-lysine-coated coverslips for 4 h. NET formation was assessed using PlaNET Green as described in Methods. [file Image_4.jpg]

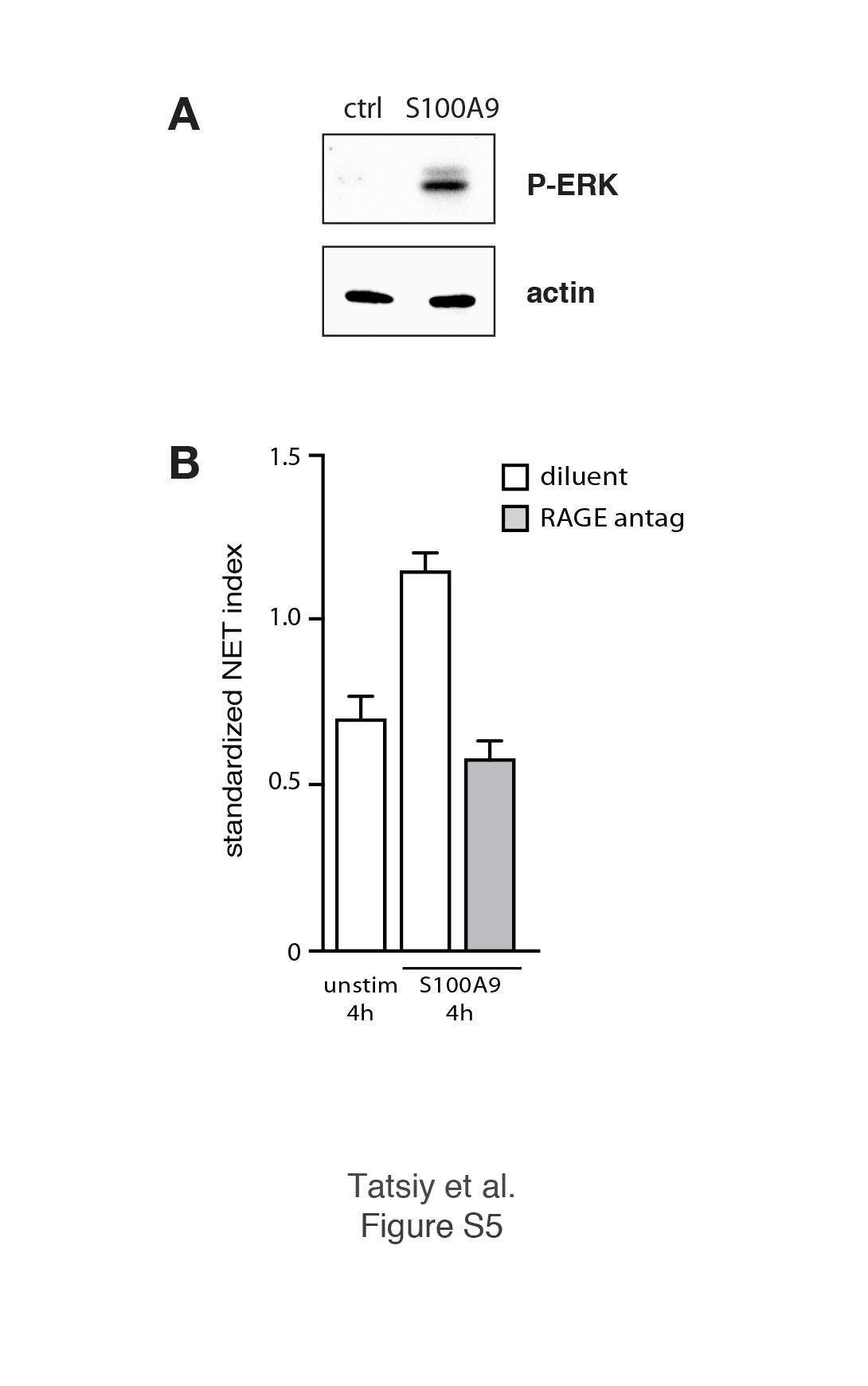

Supplement: Supplementary Figure 5 — Effect of exogenous S100A9 on neutrophil responses and involvement of RAGE. (A) Cells (3 x 10^6^/ml) were cultured for 15 min in the absence (“ctrl”) or presence of 20 µg/ml rh S100A9. Samples were then processed for SDS-PAGE analysis and immunoblot detection of P-ERK or β-actin (as a loading control); 105 cell-equivalents were loaded per lane. (B) Cells cultured on poly-L-lysine-coated coverslips were incubated in medium alone (“ctrl”), or with 20 µg/ml rh S100A9. At the 30-min time point, the RAGE antagonist FPS-ZM1 (1 µM final concentration) or its diluent (DMSO, 0.1% final concentration) was added, and neutrophils were further incubated for another 3.5 h. NET formation was assessed using PlaNET Green as described in Methods. Mean ± s.e.m. from at least 4 representative fields. [file Image_5.jpg]

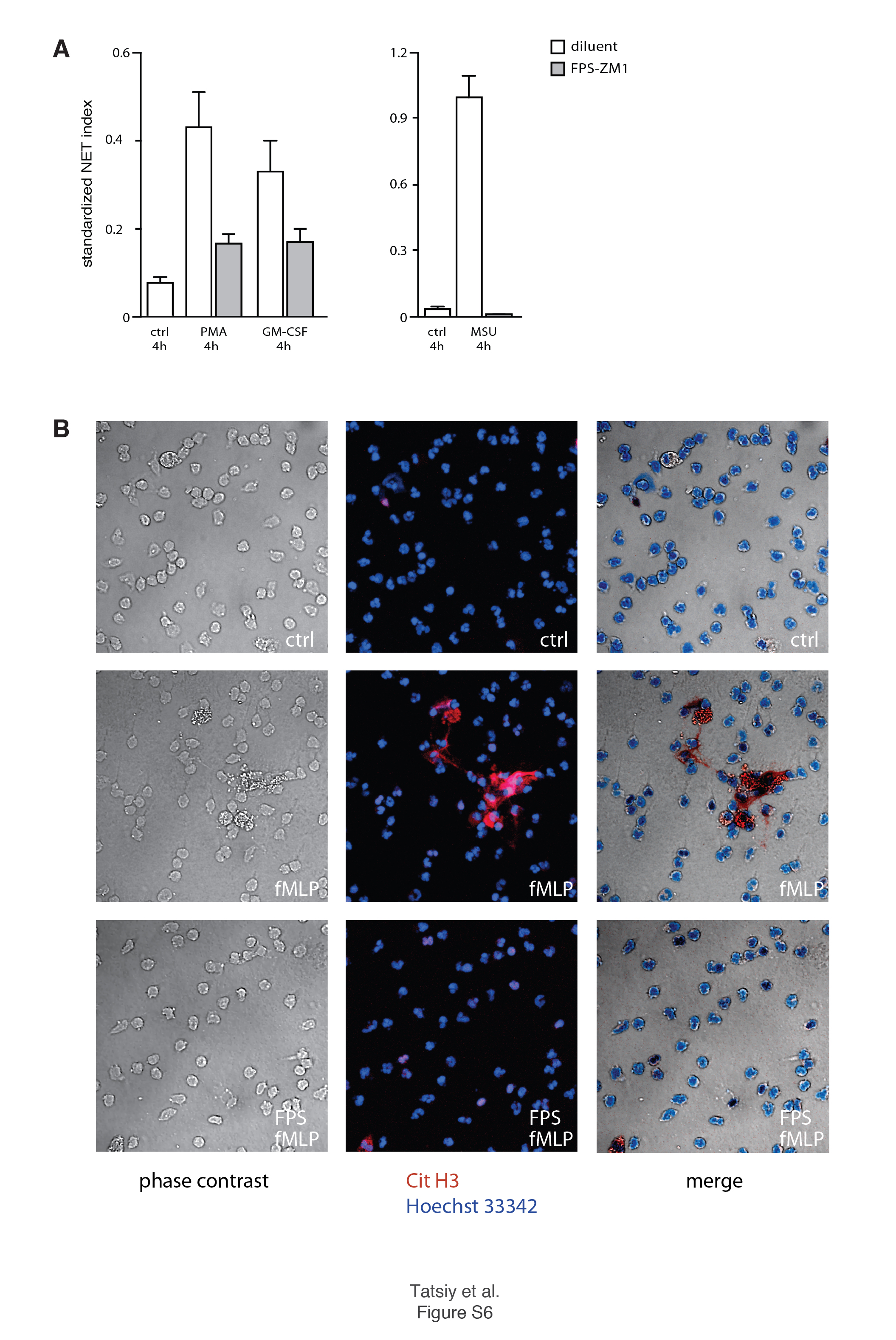

Supplement: Supplementary Figure 6 — Effect of RAGE blockade on NET induction by neutrophil agonists. (A) Cells cultured on poly-L-lysine-coated coverslips were incubated in medium alone (“ctrl”), or with either 1 nM GM-CSF, 1 mg/ml MSU, or 50 nM PMA. At the 30-min time point, the RAGE antagonist FPS-ZM1 (1 µM final concentration) or its diluent (DMSO, 0.1% final concentration) was added, and neutrophils were further incubated for another 3.5 h. NET formation was then assessed using PlaNET Green (or PlaNET Blue in the case of MSU) as described in Methods. Mean ± s.e.m. from at least 4 representative fields. (B) Neutrophils cultured on poly-L-lysine-coated coverslips were incubated as described above, albeit using 100 nM fMLP as stimulus. NET formation was assessed using Cit H3 antibodies as described before for myeloperoxidase (13). [file Image_6.jpg]
