# Supplementary material for: Early and Late Processes Driving NET Formation, and the Autocrine/Paracrine Role of Endogenous RAGE Ligands
Source: Front Immunol. 2021 Sep 20;12:675315. doi: 10.3389/fimmu.2021.675315 (PMC8488397; doi:10.3389/fimmu.2021.675315)
Supplement: Supplementary file 8 [file Table_1.pdf]

Table S1. Neutrophils were stimulated in the absence or presence of 100 U/ml TNFα for 3h; culture supernatants were collected, immunodepleted of stimulus as described under *Methods*, and processed for MS proteomics analysis. Depicted are the proteins featuring at least a 2-fold induction (TNF vs unstimulated), in two independent experiments.

**Expt 1**		**Expt 2**	
**gene name**	**fold induction**	**gene name**	**fold induction**
A6XMW0	7.34	ACTB	12.71
ACTN1	5.63	ACTG2	4.9
ACTR2	3.9	ACTN1	9.96
		ACTR2	3.34
ADSS	3.72	ADSS	3.34
ANXA11	2.36	AHSG	2.29
ANXA6	2.99	ALDOA	8.4
ARMC8	2.36	ANXA1	2.71
ARPC1B	5.98	ANXA3	2.19
		ANXA6	4.12
		APOA4	8.02
		APOB	18.17
		APOC1	3.34
		APOE	1.78
		ARG1	2.06
		ARHGAP12	2.56
		ARHGDIA	2.45
		ARHGDIB	2.23
		ARPC1B	2.56
ARPC2	6.89	ARPC2	7.24
ARPC3	3.26	ARPC3	3.34
ARPC4-TTLL3	3.26	ARPC4-TTLL3	4.12
ARPC5	2.81	B0UXW4	4.73
B0UZ83	2.73	B0UZ83	4.12
B4DQR8	2.36	B4DGP8	2.56
BASP1	2.36	B4DGQ0	2.56
BPI	8.24	B4DT30	2.56
		BTBD1	3.34
		C1QB	4.12
		C1QC	5.68
		C3	2.07
C4BPA	2.08	C4BPA	4.5
CDA	2.4	C5	2.56
CLTC	3.26	C6	2.56
CORO1A	3.99	CACNA1A	3.34
CPNE7	2.36	CAMP	5.68
CR1	2.81	CD80	3.34
CSTB	2.36	CEACAM6	2.56
		CLU	3.62
		CORO1A	4.93
		COTL1	2.56
		CPPED1	2.56
D6PXK4	6.89	D6PXK4	3.34
		DBI	2.56
DDX39B	2.36	DDX39B	4.12
		DNMBP	3.34
		E1P506	2.56
E9PMM6	14.58	E9PMM6	11.93
EFHD2	2.81	EFHD2	2.56
ENOSF1	2.36	F2	2.37

EPX	13.23	F5GXQ1	2.97
F5H3P5	7.34	F5H3P5	8.02
		F5H4W9	4.9
F6USW4	6.89	F6USW4	4.12
		FBLN5	3.34
		FBXO38	2.56
		FCGR3A	2.56
		FERMT3	3.34
		FETUB	2.56
		FGB	2.08
		FGG	4.98
		FKBP12-EXIP2	2.41
FLNA	3.87	FLNA	7.91
		FN1	3.77
G6PD	4.12	G6PD	6.46
		G8JL88	3.23
GCA	5.53	GCA	5.68
GCC2	3.72		
GDI2	3.22	GDI2	4.9
GPI	3.41	GMFG	4.12
GRN	2.31	GNB4	2.56
H0Y858	2.36	GSTP1	2.41
H3F3C	2.75	H3F3C	10.37
HIST1H2BA	2.74	HABP2	4.9
HIST1H4L;HIST1H 4K;HI...	2.79	HIST1H2BA	2.51
HK3	8.24	HK3	2.56
HNRNPA1L2	2.36	HNRNPM	2.56
		IGFALS	2.56
IGHG2	2.36	IGHG3	2.16
		IGHG4	3.23
		IGHV1-46	3.34
		IGHV3-33	2.56
		IGHV3-48	2.67
		IGHV4-59	2.56
		IGKV1-16	2.56
		IGKV1D-12	3.34
		IGKV2-30	2.06
		IGKV3-11	2.84
		IGLC1	3.19
		IGLV3-21	2.06
		IGLV3-25	4.12
IQGAP1	4.17	IQGAP1	2.56
ITGAM	2.46	ITGAM	6.46
ITGB2	2.06	ITGB2	3.34
ITIH1	5.08	ITIH1	2.42
LDHA	2.21	ITIH2	2.75
LMNB1	3.72	ITIH3	2.56
		KLKB1	2.56
		LCP1	3.34
		LDHA	2.41
		LRG1	2.56
LTA4H	10.51	LTA4H	3.96
		MACF1	3.34
		MLF1	2.56
		MMP8	6.46
MMP9	4.35	MMP9	4.9
MNDA	2.31	MSH2	4.9
MYH9	2.27	MSN	4.88
MYL6	2.6	MYH9	3.26

		NCF1	2.56
NME1	2.81	NME1	2.56
		OLFM4	2.45
P01596	3.26	P01613	4.9
P01610	3.26	P01623	2.25
P06309	3.26	P01781	2.2
P06314	2.36	PDE4D	2.56
P4HB	2.81		
PEBP1	2.81		
PFN1	2.13	PFN1	3.83
PGAM1	2.46	PGAM1	13.49
PGLS	2.81	PKM	2.27
PGM1	3.26	PLG	4.05
POU4F1	3.26	PRDM1	2.56
POU4F2	2.36	PROS1	3.62
PRG3	2.36	PZP	6.46
PRRC2C	2.36		
Q5SYT8	3.72	Q5SYT8	4.12
Q5T0H9	3.26	Q5TCC4	2.56
Q86U12	5.08	Q86U12	3.34
RAB5B	2.81		
RAB7A	3.26	RAB7A	4.9
RNH1	2.36	RAC1	2.56
S100A11	4.17	RAN	2.56
		RPIA	2.56
		S100A4	4.12
S100A6	3.44	S100A6	2.93
		SERPINA1	2.56
		SERPINA3	3.57
SERPINB1	2.73	SERPINB1	4.24
SERPINB10	7.34	SERPIND1	8.8
SH3BGRL	2.81		
SPTBN1	2.36		
TAGLN2	2.81		
TLN1	5.08	TLN1	3.34
TUBB3	2.36	TUBB3	4.12
TXN	3.72		
U2SURP	2.81	UMPS	2.56
UBE2C	2.36		
VASP	3.26	VASP	4.9
VAT1	2.36		
VCL	7.79	VCL	4.12
		ZC3H12B	2.56
