# Supplementary material for: Early and Late Processes Driving NET Formation, and the Autocrine/Paracrine Role of Endogenous RAGE Ligands
Source: Front Immunol. 2021 Sep 20;12:675315. doi: 10.3389/fimmu.2021.675315 (PMC8488397; doi:10.3389/fimmu.2021.675315)
Supplement: Supplementary file 9 [file Table_2.pdf]

Table S2. Unstimulated neutrophils were cultured for either 30 min or 3h; supernatants were collected and processed for MS proteomics analysis as described under *Methods*,. Depicted are the proteins featuring at least a 2-fold induction (unstimulated 3h vs unstimulated 30 min), in two independent experiments.

**Expt 1**		**Expt 2**	
**gene name**	**fold induction**	**gene name**	**fold induction**
A6XMW0	7.46	A6XMW0	3.46
		ACTN1	3.77
ACTR2	3.96	ACTR2	7.35
ADSS	3.77	ADSS	3.54
		ALDOC	4.38
		APCS	2.27
		ARG1	3.12
ARMC8	2.38	ARMC8	2.27
ARPC1B	3.04	ARPC3	3.12
		ARPC5	2.69
		B0YJCR4	20.89
B4DQR8	2.38	B4DQR8	2.27
B4DZX3	2.38	B4DZX3	2.27
BASP1	2.38	BASP1	2.27
BPI	8.39	BPI	7.77
		CAT	3.4
		CDA	2.26
CHI3L1	2.85	CHI3L1	2.69
CHIT1	3.77	CLIC1	2.27
CLTC	3.31	CLTC	3.12
CPNE7	2.38	CPNE7	2.27
CPPED1	2.85	CPPED1	2.69
CR1	2.85	CR1	2.69
		CR9J8S3	3.12
		CR9JPV4	2.27
		CR9JUM1	4.81
		CR9JV37	4.38
		CR9JV77	2.27
CSTB	2.38	CSTB	2.27
D6PXK4	3.5	D6PXK4	6.5
DDX39B	2.38	DDX39B	2.27
DOT1L	3.31	DOT1L	3.12
		E7EMCR6	5.65
E9PMM6	7.42	E9PMM6	4.56
EEF1G	2.38	EEF1G	2.27
ELANE	3.77	ELANE	2.7
ENO1	2.48	ENO1	3.54
ENOSF1	2.38	ENOSF1	2.27
F6USW4	2.33	F6USW4	3.25
FKBP12-EXIP2	2.03		
FLNA	3.94	FLNA	2.91
FTL	2.38	FTL	2.27
G6PD	2.1	G6PD	3.88
GCA	5.62	GCA	5.23
GCC2	3.77	GCC2	3.54
GDI2	3.27	GDI2	3.04
GPI	3.47	GPI	9.6
		GSR	2.27
		GYG1	2.31
H0Y858	2.38	H0Y858	2.27
		H0YCRR7	2.27

H7BYC5	3.31	H7BYCR5	3.12
		H7CR0A3	3.12
		H7CR1M3	3.12
		H7CR3F9	6.5
		H7CR5R1	11.58
HBA2	2.38	HBA2	2.27
HBD	2.85	HBD	2.69
HK3	2.1	HK3	3.88
HSPA5	2.38	HSPA5	2.27
HSPA7	3.31	HSPA7	3.12
IGHD	2.38	IGHD	2.27
IQGAP1	4.23	IQGAP1	3.96
ITGB2	2.8		
		J3QLCR9	16.65
		LCP1	3.15
LDHA	3.94	LDHA	4.85
		LMNB1	3.54
LSP1	2.38	LSP1	2.27
LTA4H	3.56	LTA4H	3.3
MAP2K4	2.85	MAP2K4	2.69
MMP9	2.95	MMP9	4.1
MSH2	3.77	MSH2	3.54
		MYH9	3.2
NCF1	3.77	NCF1	3.54
NME1	2.85	NME1	2.69
OLFM4	2.58	OLFM4	2.4
		P06309	3.12
		P0CR0S5	5.23
		P0CRG05	8.62
		P4HB	2.69
PARK7	2.38	PARK7	2.27
PEBP1	2.85	PEBP1	2.69
		PFN1	2.16
		PGD	2.45
PGLS	2.85	PGLS	2.69
		PGM1	3.12
PLBD1	3.31	PLBD1	3.12
		PNP	3.46
POU4F2	2.38	POU4F2	2.27
PRG3	2.38	PRG3	2.27
PRRC2C	2.38	PRRC2C	2.27
		Q567Q0	2.49
Q5SYT8	3.77	Q5SYT8	3.54
Q5T0H9	3.31	Q5T8M7	2.27
Q86U12	5.15	Q86U12	4.81
		Q96CR19	2.69
RAB5B	2.85	RAB5B	2.69
RAB7A	3.31		
RNH1	2.38	RPIA	2.27
S100A12	2.08	S100A12	6.4
		S100A6	2.17
S100P	2.81	S100P	5.23
SERPINB10	7.46	SERPINB10	6.92
SH3BGRL	2.85	SH3BGRL	2.69
SH3BGRL3	2.03	SNX33	3.54
SNX33	3.77		
SPTBN1	2.38	SPTBN1	2.27
TAGLN2	2.85	TAGLN2	2.69
TLN1	2.58		

TPI1	2.11	TPI1	3.5
TSPAN14	2.38	TSPAN14	2.27
TXN	3.77		
U2SURP	2.85	U2SURP	2.69
UBE2C	2.38		
VAT1	2.38		
VCL	3.96	VCL	3.67
